# A Global Nutritional Tool for Monitoring Westernized Dietary Transition: Validation of the Westernized Diet Index Using a Large Population Sample and Biomarkers of Metabolic Health

**DOI:** 10.3390/nu18020349

**Published:** 2026-01-21

**Authors:** Farhad Vahid, Reza Homayounfar, Mojtaba Farjam, Torsten Bohn

**Affiliations:** 1 Nutrition and Health Research Group, Department of Precision Health, Luxembourg Institute of Health, L-1445 Strassen, Luxembourg; farhad.vahid@lih.lu; 2 National Nutrition and Food Technology Research Institute (WHO Collaborating Center), Faculty of Nutrition Sciences and Food Technology, Shahid Beheshti University of Medical Sciences, Tehran 1981619573, Iran; r_homayounfar@yahoo.com; 3 Noncommunicable Diseases Research Center, Fasa University of Medical Sciences, Fasa 7461686688, Iran; farjam.phd@gmail.com

**Keywords:** noncommunicable diseases, dietary patterns, cardiometabolic biomarkers, public health nutrition, obesity, insulin resistance

## Abstract

Background: Dietary transitions toward Westernized patterns (WDPs) (high in processed foods, sugars, and fats) pose a global public health challenge. The Westernized Diet Index (WDI) measures adherence to these patterns. However, its validity with respect to metabolic biomarkers warrants thorough evaluation for use in epidemiological and clinical research. Objectives: This study validates the WDI using metabolic biomarkers (including anthropometrics, blood pressure, fasting blood glucose (FBG), triglycerides, HDL-c, LDL-c, and total cholesterol), examines its association with metabolic syndrome (MetS), and compares scoring methods to identify the most effective measure of WDPs adherence. Methods: Data from 10,146 participants in the Fasa Adult Cohort Study (FACS) were used. We calculated the WDI using global (WDI-G) and population (WDI-P) Z scores and food group (WDI-FG)-based algorithms. Validation employed logistic and linear regression, ROC (receiver operating characteristic) curves, Youden’s index, and k-means clustering. Results: All WDI scoring methods (across all methods, higher scores indicated lower adherence to WDPs) demonstrated a strong, significant association with all three MetS definitions (WHO, NCEP: ATPIII, and IDF) and nearly all investigated metabolic biomarkers. In fully adjusted logistic models, WDI Global (WDI-G) (OR: 0.23) and WDI Food Groups (WDI-FG) (OR: 0.26) were significantly associated with MetS (based on the WHO definition). Also, in fully adjusted linear regression models, a 10% increase (reflecting lower adherence to WDPs) in the WDI-G score (range: −2.03 to 1.11) was significantly associated with a 3.96 mg/dL reduction in FBG and a 2.61 cm reduction in waist circumference. Additionally, ROC curves (AUC: 0.57–0.61) demonstrated that WDI predicts MetS with moderate accuracy. The strongest associations were observed with population-based scoring. In addition, based on comparative performance, WDI-G, WDI-P, and WDI-FG appear most suitable for cross-population, within-cohort, and mechanistic or intervention-focused research, respectively. Conclusions: The WDI shows promise as a nutritional tool for assessing adherence to WDPs and exploring associations with metabolic health outcomes, including MetS. These findings suggest that the WDI may be useful in future dietary, public health, and clinical research, although further validation in diverse populations is warranted.

## 1. Introduction

The increasing prevalence and burden of noncommunicable diseases (NCDs) pose a major global public health challenge [1]. NCDs, including cancer, cardiovascular diseases (CVDs), type 2 diabetes (T2D), obesity, and metabolic syndrome (MetS), account for approximately 80% of global mortality and morbidity, with CVDs alone being responsible for over 17.9 million deaths annually [1,2]. A main driver of many NCDs is obesity, with obesity rates having tripled worldwide since 1975, with more than 1.9 billion adults being classified as having overweight or obesity [3]. MetS, a cluster of conditions that increases the risk of CVDs, stroke, and T2D, has seen a significant rise in prevalence, reaching an average global prevalence of 28.2% in adults when the International Diabetes Federation (IDF) definition is applied [4]. In the US, the prevalence of MetS among adults aged 20 years or older increased from 37.6% in 2011–2012 to 41.8% in 2017–2018 [4].

A significant proportion of this disease burden is attributable to modifiable lifestyle factors, particularly poor dietary habits [5]. In 2019, dietary risks were estimated to contribute to 8 million deaths and 187 million disability-adjusted life years (DALYs) globally, making unhealthy diets one of the leading risk factors for premature mortality and disease burden [5]. Research has shown that dietary patterns have a profound influence on health outcomes, particularly in the context of NCDs such as CVD [6], MetS [7], and cancers [8], among others. In recent decades, the adoption of Westernized diets (WDs), characterized by a high intake of processed foods, red meats, refined sugars, and fats, and a low intake of dietary fiber, vitamins, and minerals, has become a global phenomenon [9,10]. This shift is particularly evident in non-Western countries, where urbanization and globalization have led to rapid dietary transitions that have significantly altered the nutritional landscape and increased the risk of metabolic disorders and NCDs.

Beyond individual health risks, the global shift toward Westernized dietary patterns (WDPs) also has broader implications for food systems and sustainability [11]. The demand for highly processed, animal-based, and resource-intensive foods is reshaping agricultural production, trade, and supply chains, with consequences for environmental sustainability, equity, and global food security [12]. Understanding and quantifying adherence to WDs is therefore critical not only for linking dietary transitions to metabolic health but also for informing food system policies and interventions aimed at mitigating the dual burden of chronic disease and unsustainable food consumption. Conversely, adherence to traditional and health-promoting dietary patterns, such as the Mediterranean diet (MD), has been extensively associated with a reduced risk of NCDs, including MetS [7], among others. The protective effects of the MD are largely attributed to a high consumption of nutrient-dense foods, such as fruits, vegetables, whole grains, and unsaturated fats, as well as its rich profile of secondary bioactive plant metabolites, including antioxidants, which have been shown to modulate a variety of cellular and metabolic pathways, enhancing insulin sensitivity and reducing systemic inflammation [7], a hallmark of many NCDs [13].

A major challenge has been the limited availability of reliable and valid nutritional assessment tools to comprehensively evaluate dietary patterns/habits. In recent decades, studies have sought to develop reliable and valid methods, such as dietary indices, for assessing overall nutritional quality. These methods evaluate dietary patterns and adherence to guidelines and investigate the relationships between these assessments and health outcomes. Well-established examples that have been widely utilized and whose validity has been assessed in numerous studies include the Dietary Inflammatory Index (DII), Dietary Antioxidant Index (DAI), and the Mediterranean Diet Score (MDS) [14,15,16,17,18,19]. However, each of these indices has inherent limitations, including the exclusion of specific food groups or essential nutrients, as well as non-nutrient but bioactive compounds such as polyphenols and other phytochemicals [15,20].

Additionally, many indices lack flexibility in their application, often requiring comprehensive, detailed dietary data (e.g., the DII, which has up to 45 elements) that may not always be available, thereby limiting their utility in certain research settings [15,17,20]. Some indices, such as the Dietary Diversity Score (DDS) and the Food Variety Score (FVS), rely solely on qualitative categorizations of the diet (e.g., healthy, unhealthy), which reduces the accuracy of the analyses rather than providing a continuous or quantitative measure of adherence [16]. Additionally, some indices focus primarily on nutrient density (e.g., the DAI [6,18]), while others emphasize certain food-group patterns (e.g., the MDS [16,19]), making them more suitable for assessing specific dietary components rather than providing a comprehensive evaluation of overall diet quality.

However, none of the existing dietary indices have explicitly focused on assessing adherence to the WDPs, despite their well-established association with adverse health outcomes and their increasing global prevalence [9,21]. The Westernized Diet Index (WDI) is a novel nutritional tool designed and developed [22] to quantify adherence to the WD, offering a standardized method to assess diet quality in both Western and non-Western populations. While the WDI has shown promise as a measure of dietary adherence [22], its validity, particularly in relation to metabolic health, has yet to be comprehensively evaluated. Validation against metabolic biomarkers, which capture the physiological consequences of dietary transitions, is crucial for establishing the relevance of the WDI in relating diet quality to metabolic health. This study addresses this gap by evaluating the WDI’s validity through its association with key cardiometabolic biomarkers and by providing a framework for its application in public health and clinical practice.

## 2. Materials and Methods

### 2.1. Population

The Fasa Adult Cohort Study (FACS) is an ongoing, longitudinal, prospective cohort study investigating adults aged 35 years and older in Fasa, Iran. Participants underwent comprehensive assessments, including anthropometric measurements, medical history, demographic data, and dietary intake evaluation, using a validated 125-item food frequency questionnaire (FFQ). Detailed information on the study design, data collection protocols, and methodology has been described elsewhere [23]. Out of the 10,146 participants initially enrolled in the study, 10,121 (99.75%) had complete dietary data. Among those with available dietary data, 9486 participants (93.7%) had complete MetS data and were retained for analysis, while 635 participants (6.3%) were excluded due to missing MetS data. Consequently, the final analytical sample comprised 9486 participants, accounting for 93.5% of the total study population (Supplementary Figure S1).

All participants were provided with detailed information regarding the study’s objectives and purpose, and they gave written informed consent. The study protocol was approved by the Ethics Committee of Fasa University of Medical Sciences (Approval Code: IR.FUMS.REC.1402.095) and was conducted in accordance with the principles outlined in the Declaration of Helsinki.

### 2.2. Variables and Confounders

The following variables were extracted from the FACS database: dietary intake data, including total energy intake, food groups, macro-and micronutrients, and non-nutients calculated by linking FFQ data to the USDA database using Nutritionist IV software (version 7.0; *N*-Squared Computing, Salem, OR, USA), age (years), sex (women/men), current smoking status (yes/no), anthropometric data included body mass index (BMI) (kg/m^2^), waist circumstance (WC) (cm), hip circumstance (HC) (cm), waist to hip ratio (WHR), physical activity assessed by the International Physical Activity Questionnaire (IPAQ) and expressed in the metabolic equivalent of tasks (METs), systolic and diastolic blood pressure (SBP and DBP) (mmHg), pulse rate (bpm), mean arterial pressure (MAP = DBP+1/3(SBP−DBP)) (mmHg), fasting blood glucose (FBG) (mg/dL), triglycerides (TG) (mg/dL), total cholesterol (mg/dL), low-density lipoprotein cholesterol (LDL-c) (mg/dL), and high-density lipoprotein cholesterol (HDL-c) (mg/dL).

### 2.3. Defining Metabolic Syndrome (MetS)

In this study, we employed three distinct definitions to define MetS, including those established by the World Health Organization (WHO) [24,25], the National Cholesterol Education Program’s Adult Treatment Panel III (NCEP: ATPIII) [26,27], and the International Diabetes Federation (IDF) [28]. These definitions were selected to ensure a comprehensive assessment of MetS across different diagnostic criteria. The relevant cutoff values and diagnostic components utilized for classifying participants according to each of these MetS definitions are presented in Supplementary Table S1, outlining the specific criteria for each definition, highlighting the components required for MetS diagnosis, which include measurements of central obesity, blood pressure, triglyceride levels, HDL-c, and blood glucose status.

### 2.4. Calculation of the Westernized Diet Index (WDI)

In this study, several methods were suggested to calculate the previously developed WDI [22], with the aim of assessing the degree of adherence to a WD. The underlying concept of the WDI was recently published [22]; the index is based on 30 food groups/items. Supplementary Figure S2 provides a practical decision guide for selecting the appropriate WDI calculation method based on study objectives and data availability: global Z-score–based indices (WDI-G and related variants) are recommended for cross-population comparisons and international studies; population-based indices (WDI-P variants) are better suited for within-cohort analyses emphasizing relative dietary contrasts; food group–based (WDI-FG) and individual-component (WDI-I) approaches are most appropriate for mechanistic or intervention-focused research targeting specific dietary components. The detailed computational methods and algorithms are outlined below (in all methods, higher scores indicate lower adherence to WDPs).

#### 2.4.1. WDI Based on Global Z-Scores

a. WDI Global (WDI-G)

The WDI-G was calculated by standardizing dietary intake data using global Z-scores (global mean of the food (item/group) intake and their standard deviations (SD)) (Supplementary Table S2). This was achieved by calculating global Z-scores ((participant’s intake—global mean intake)/global SD) for each food component/item based on the mean and SD of the intake across the global population, for which data were taken from several sources, e.g., world food consumption database (FAO/WHO GIFT) [29,30], which included both Western and non-Western populations (Supplementary Table S2). These Global Z-scores were then multiplied by their corresponding WDI coefficients derived from a prior study [22] (Supplementary Table S2).

Specifically, for each component/item, a Global Z-score was computed as:

$$Global\;Z-score\;for\;food\;item\;n1=\frac{participant’s\;intake\;of\;food\;item\;n1-Global\;mean\;intake\;of\;food\;item\;n1}{Global\;SD\;of\;food\;item\;n1}$$

and this was repeated for all 29 food items and divided by the number of food items (*n* = 29), i.e., as follows:

*WDI-G* = ((*Global Z-score for food item n*1 × *coefficient of food item n*1) + (*Global Z-score for food item n*2 × *coefficient of food item n*2) +    + (*Global Z-score for food item n*28 × *coefficient of food item n*28) + (*Global Z-score for food item n*29 × *coefficient of food item n*29))/29

For the final WDI-G score, the global Z-scores for 29 out of 30 food components/items were summed and divided by 29. It should be noted that no data were available for dietary supplements, and as such, these were excluded from the calculation.

For food components/groups in the index [22] e.g., vitamins and minerals group, summarizing various constituents such as vitamins, minerals, and secondary plant metabolites, with different units of intake measurement, individual global Z-scores for each item were calculated separately, then individual Z-scores summed up and divided by the number of items in the group, providing one single Z-score for that food component/group, and then that Z-score for that food component/group was included in the formula. Differing from the previous group, for other food components/groups, such as fruit and vegetable groups, whose constituents within the group had the same units, the global Z-scores were calculated by summing the individual items within the group and standardizing them accordingly.

b. WDI Global Centralized (WDI-GC)

The WDI-GC is a variation of the WDI-G. To minimize the effect of extremely high or low intakes, which are common in right-skewed dietary data [31], the WDI coefficients were converted to a percentile score using Fractional Rank as % (see Supplementary Table S2 for the Fractional Rank of the WDI coefficients). To obtain a symmetrical distribution centered on 0 (null) and bounded between −1 (maximal adherence to WD) and +1 (minimal adherence to WD), each percentile score was doubled, and one was subtracted. This centralization process enabled a more centralized scoring method to account for variations in dietary intakes across different populations.

*WDI-GC* = ((*Global Z-score for food item n*1 × *Centralized coefficient of food item n*1) + (*Global Z-score for food item n*2 × *Centralized coefficient of food item n*2) +       + (*Global Z-score for food item n*28 × *Centralized coefficient of food item n*28) + (*Global Z-score for food item n*29 × *Centralized coefficient of food item n*29))/29

c. WDI Global Standardized (WDI-GS)

The WDI-GS is also a variation of the WDI-G. Following the calculation of the WDI-G, the WDI-GS was derived by further standardizing the WDI-G using the population’s Z-score. This additional step ensured that dietary intake values were adjusted to the population distribution, providing a more refined representation of adherence to the WD.

*WDI-GS* = (*Participant’s WDI-G* − *population’s mean of the WDI-G*)/*population’s SD for the WDI-G*

#### 2.4.2. WDI Based on Population Z-Scores

a. WDI Population (WDI-P)

The WDI-P was calculated similarly to the WDI-G, but using population-specific data. The means and SDs for food components/items within the population cohort were used to standardize the intake data into population Z-scores. These Z-scores were then multiplied by the corresponding WDI coefficients (Supplementary Table S2). Similar to WDI-G, for certain groups with different units of intake, such as vitamins, minerals, and secondary plant metabolites, population Z-scores for individual items were calculated, summed, and averaged. Other food groups were similarly handled by calculating population Z-scores for grouped items. In the final WDI-P, the Z-scores for 29 out of 30 food components/items were summed and divided by 29.

Specifically, for each component/item, a population Z-score was computed as:

$$Population\;Z-score\;for\;food\;item\;n1=\frac{participant’s\;intake\;of\;food\;item\;n1-Population\;mean\;intake\;of\;food\;item\;n1}{Population\;SD\;of\;food\;item\;n1}$$

repeated for all 29 food items and divided by the number of food items (*n* = 29):

*WDI-P* = ((*Population Z-score for food item n*1 × *coefficient of food item n*1) + (*Population Z-score for food item n*2 × *coefficient of food item n*2) +        + (*Population Z-score for food item n*28 × *coefficient of food item n*28) + (*Population Z-score for food item n*29 × *coefficient of food item n*29))/29

b. WDI Population Centralized (WDI-PC)

The WDI-PC is a variation of the WDI-P. Similar to the WDI-C, to minimize the effect of extremely high or low intakes, which are common in right-skewed dietary data [31], the WDI coefficients were converted to a percentile score using Fractional Rank as % (see Supplementary Table S2 for the Fractional Rank of the WDI coefficients). To obtain a symmetrical distribution centered on 0 (null) and bounded between −1 (maximally adherence to WD) and +1 (minimally adherence to WD), each percentile score was doubled, and one was subtracted.

*WDI-PC* = ((*Population Z-score for food item n*1 × *Centralized coefficient of food item n*1) + (*Population Z-score for food item n*2 × *Centralized coefficient of food item n*2) +         + (*Population Z-score for food item n*28 × *Centralized coefficient of food item n*28) + (*Population Z-score for food item n*29 × *Centralized coefficient of food item n*29))/29

c. WDI Population Standardized (WDI-PS)

The WDI-PS follows the WDI-P approach, but similar to WDI-S, it incorporates an additional step of standardization. After deriving the population-specific Z-scores, these were standardized using the overall population Z-score to ensure that the data were consistent with population-specific dietary intake distributions.

*WDI-PS* = (*Participant’s WDI-P* − *population’s mean of the WDI-P*)/*population’s SD for the WDI-P*

#### 2.4.3. WDI Individual (WDI-I)

In a completely different approach, the intake of all food components/items was multiplied by their respective WDI coefficients (Supplementary Table S2) [22], including individual vitamins, minerals, and secondary plant metabolites. Subsequently, the population Z-scores for these weighted items (WDI coefficients * individual food components/items) were calculated and averaged by dividing by the sum of the total number of components/items (57 items). The key distinction between this method and the previous ones (WDIG and WDI-P) is that, in earlier approaches, global/population Z-scores of the food components/items were calculated first and then multiplied by the WDI coefficients. In contrast, this method first multiplies the intake values by the WDI coefficients and then calculates the Z-scores (in this study, only population Z-scores).

*WDI-I* = ((*population Z-scores of (food item n*1 × *coefficient of food item n*1)) + (*population Z-scores of (food item n*2 × *coefficient of food item n*2)) +        + (*population Z-scores of (food item n*56 × *coefficient of food item n*56)) + (*population Z-scores of (food item n*57 × *coefficient of food item n*57)))/57

#### 2.4.4. WDI Food Groups (WDI-FG)

The WDI-FG categorizes food components into 18 distinct food groups, which include whole grains, refined grains, legumes, nuts and seeds, oils, refined fats, soft drinks, coffee/tea/waters, processed foods, fruits, vegetables, red meat, processed meat, white meat, fish, dairy, diet drinks, and alcoholic drinks. Similar to WDI-I, for the WDI-FG, each food group’s intake is multiplied by the corresponding WDI coefficients (Supplementary Table S2) [22], and the Z-scores for each food group are calculated. The final score is the sum of the Z-scores for each group, divided by the total number of food groups (18 items).

*WDI-FG* = ((*population Z-scores of (food group n*1 × *coefficient of food group n*1)) + (*population Z-scores of (food group n*2 × *coefficient of food group n*2)) +        + (*population Z-scores of (food group n*17 × *coefficient of food group n*17)) + (*population Z-scores of (food group n*18 × *coefficient of food item n*18)))/18

### 2.5. Statistical Analysis

#### 2.5.1. Data Management and Descriptive Analyses

Data management and statistical analyses were performed using IBM SPSS Statistics version 25.0 (IBM Corp, Armonk, NY, USA) and R version 4.4.1 (R Foundation for Statistical Computing, Vienna, Austria). All tests were two-tailed, and statistical significance was set at a *p*-value < 0.05.

Assumptions for all statistical analyses were carefully checked. The normality of continuous variables was assessed using visual inspection of Q-Q plots. Homogeneity of variances was evaluated using Levene’s test, which indicated no significant differences in variances across groups, suggesting that the assumption of homogeneity of variances was satisfied. Multicollinearity was assessed using variance inflation factors (VIFs), and no issues were detected, as all VIFs were below the threshold. Linearity and independence of observations were confirmed for all regression analyses by examining scatter plots of residuals against predicted values and performing the Durbin–Watson test for autocorrelation. The residuals of the linear regression models were assessed for homoscedasticity and normality through visual inspection of residuals-versus-fitted-values plots and Q-Q plots, respectively, and no violations were found.

Additionally, the Durbin–Watson statistic was checked to assess residual independence, confirming that the assumption of no autocorrelation was satisfied. For the Receiver Operating Characteristic (ROC) curves and AUC calculations, the independence of observations was ensured. The monotonic association between the true positive rate (sensitivity) and the false positive rate (1-specificity) was also satisfied. For Youden’s index (J index), the assumptions of independence of observations and appropriate sensitivity and specificity calculations were conducted based on the classifier’s performance.

For the descriptive analyses, independent-samples *t*-tests were performed to compare the means and SDs of WDI scores and key biomarkers across different MetS classifications and K-means clusters. This allowed an assessment of how adherence to a WD (as measured by WDI) varied with metabolic health status and clustering patterns based on metabolic biomarkers. For each group (MetS vs. non-MetS and healthy vs. unhealthy clusters), means and SDs were compared using independent-samples *t*-tests for WDI scores and for relevant biomarkers, including FBG, cholesterol levels, triglycerides, LDL-c, HDL-c, WC, HC, WHR, BMI, SBP, DBP, PR, and MAP.

#### 2.5.2. Logistic Regression Models

Crude and adjusted logistic regression models were used to estimate the odds ratios (ORs) and 95% confidence intervals (CIs) for the association between different WDI scoring methods and different MetS classifications. The crude models provided unadjusted estimates, while the adjusted models accounted for potential confounders, including age and sex. Furthermore, fully adjusted models incorporated additional variables, such as physical activity (measured in METs), smoking status, and total energy intake, which could influence the likelihood of MetS. By including these additional factors, the fully adjusted models provided a more nuanced view of the WDI’s association with MetS, accounting for key lifestyle and health-related variables.

#### 2.5.3. Linear Regression Models

Linear regression models were used to examine the association between WDI and continuous metabolic biomarkers, including FBG, TG, cholesterol levels, and blood pressure. These models were initially run in their crude form to identify unadjusted associations, followed by adjustment for age, sex, and other potential confounders, such as physical activity, smoking, and total energy intake. The fully adjusted models provided more precise estimates of the strength and direction of the association between WDI and each metabolic biomarker, allowing for a clearer understanding of how adherence to a WD influences various metabolic processes. The regression coefficients (β) and 95% CIs were used to quantify the strength and direction of the association, providing insights into the potential biological mechanisms linking the WDI to health outcomes.

#### 2.5.4. Receiver Operating Characteristic (ROC) Curves

Receiver operating characteristic (ROC) curves were constructed to assess the discriminatory power of WDI in identifying individuals with MetS based on different definitions (WHO, ATPIII, IDF). The ROC curves provided an effective means to evaluate the WDI’s ability to distinguish between individuals with and without MetS, helping establish its clinical utility as a tool for identifying individuals at risk of MetS. The area under the curve (AUC) was calculated using the nonparametric trapezoidal (Wilcoxon–Mann–Whitney) method, as implemented in SPSS and R, for each WDI scoring method, with a higher AUC indicating better discriminatory power. Optimal cutoff points for each scoring method were identified using Youden’s index, which maximizes the sum of sensitivity and specificity, providing a balance between correctly identifying individuals with MetS and avoiding false positives. This analysis allowed for the determination of the most appropriate WDI thresholds for use in clinical and research settings.

#### 2.5.5. K-Means Clustering

Unsupervised k-means clustering was applied to explore the association between WDI and metabolic biomarkers in greater depth. Unlike regression or ROC analyses, which quantify linear associations or predictive performance for predefined outcomes, clustering identifies naturally occurring patterns within the data without imposing prior assumptions. The clustering technique grouped participants into two distinct clusters: a “healthy” group and an “unhealthy” group, based on their overall metabolic profiles. These empirically derived clusters capture heterogeneity in metabolic health that may not be apparent from individual biomarker associations alone. By identifying these distinct clusters, the analyses offered valuable insights into how adherence to a WD is associated with different metabolic health outcomes, providing an additional layer of construct validation. The final cluster centers were compared across different WDI scoring methods to assess how well the WDI aligned with distinct patterns of metabolic health. This approach not only complements the regression and ROC analyses but also offers insights into the broader patterns linking dietary adherence to the WD with metabolic health, highlighting potential subgroups at higher risk for adverse outcomes. Unlike the predefined MetS definitions, k-means clustering was applied as an unsupervised, data-driven approach to identify natural groupings of metabolic health based on continuous biomarkers, thereby providing complementary construct validation of the WDI independent of diagnostic thresholds.

## 3. Results

### 3.1. Descriptive Results

The baseline characteristics of the participants are presented in Table 1. The comparison (mean ± SD) of the WDI and biomarkers across participants categorized by MetS definitions (WHO, ATPIII, IDF) is presented in Table 1. In addition to comparisons based on established MetS definitions, k-means clustering was used to identify empirically derived metabolic health profiles, allowing assessment of whether WDI scores differed across data-driven ‘healthy’ and ‘unhealthy’ clusters. Across all MetS definitions, individuals with MetS consistently showed lower WDI scores compared to those without MetS, with statistically significant differences (*p* < 0.001 for all comparisons), except for the WDI individual in the IDF definition (*p*-value = 0.62). Specifically, the mean WDI values were notably lower in individuals with MetS (ranging from −0.32 to 0.96) than in those without MetS (ranging from −0.13 to 0.98), indicating greater adherence to the WD in participants with MetS. In terms of biomarker measurements, and as expected by its definition, individuals with MetS had statistically significantly higher values for WC, HC, WHR, BMI, SBP, DBP, FBG, TG, and total cholesterol (all *p* < 0.001).

The WDI methods exhibited varying ranges (minimum–maximum): WDI-G ranged from −2.03 to 1.11 (3.14 units), WDI-GC from −3.06 to 3.21 (6.27 units), WDI-GS from −9.39 to 6.20 (15.59 units), WDI-P from −0.36 to 0.42 (0.78 units), WDI-PC from 0.27 to 1.83 (1.56 units), WDI-PS from −7.27 to 8.77 (16.04 units), WDI-I from −3.59 to 1.07 (4.66 units), and WDI-FG from −1.91 to 2.81 (4.72 units) (means ± SD are presented in Table 1).

**Table 1:** Comparison (mean ± SDs or (n%)) of baseline characteristics, WDI scores, and biomarkers by MetS and K-means clusters.

Variables	Total = 9486	MetS (WHO)			MetS (ATPIII)			MetS (IDF)			K-Means Cluster **		
		No = 8733 (92.1%) ^†^	Yes = 753 (7.9%) ^†^	*p*-Value	No = 7672 (80.9%) ^†^	Yes = 1814 (19.1%) ^†^	*p*-Value	No = 7727 (81.5%) ^†^	Yes = 1759 (18.5%) ^†^	*p*-Value	Healthy = 8282 (87.3%)	Unhealthy = 1202 (12.7%)	*p*-Value
Baseline characteristics													
Age (years)	48.9 ± 9.4	48.5 ± 9.4	54.0 ± 8.4	<0.001	48.3 ± 9.4	51.6 ± 9.2	<0.001	48.3 ± 9.7	51.6 ± 9.2	<0.001	49.0 ± 9.5	48.7 ± 90.1	0.402
Sex (women)	52.92 (55.8%)	4738 (89.5%)	554 (10.4%)	<0.001	3911 (73.9%)	1381 (26.1%)	<0.001	3852 (72.8%)	1440 (27.2%)	<0.001	4686 (88.5%)	605 (11.4%)	<0.001
METs	41.4 ± 11.2	41.7 ± 11.4	38.1 ± 7.7	<0.001	42.1 ± 11.7	38.5 ± 8.1	<0.001	42.1 ± 11.7	38.2 ± 7.6	<0.001	41.5 ± 11.3	40.4 ± 10.8	<0.001
Smoking status (no)	8711 (91.8%)	8020 (92.06%)	691 (7.93%)	0.945	7006 (80.4%)	1705 (19.6%)	<0.001	7051 (80.9%)	1660 (19.1%)	<0.001	7615 (87.4%)	1094 (12.6%)	0.284
Total energy intake (kcal)	2922 ± 1134	2943 ± 1129	2686 ± 1159	<0.001	2958 ± 1141	2772 ± 1090	<0.001	2963 ± 1142	2742 ± 1081	<0.001	2907 ± 1133	3030 ± 1138	<0.001
WC (cm)	93.2 ± 11.8	92.5 ± 11.6	101.9 ± 10.6	<0.001	91.0 ± 11.2	102.6 ± 9.4	<0.001	90.8 ± 11.0	104.0 ± 8.74	<0.001	92.5 ± 11.9	98.1 ± 9.8	<0.001
HC (cm)	99.6 ± 8.9	99.2 ± 8.7	103.5 ± 9.6	<0.001	98.4 ± 8.6	104.4 ± 8.5	<0.001	98.2 ± 8.5	105.5 ± 9.3	<0.001	99.2 ± 9.0	101.9 ± 7.8	<0.001
WHR	0.93 ± 0.06	0.93 ± 0.04	0.98 ± 0.05	<0.001	0.92 ± 0.06	0.98 ± 0.05	<0.001	0.92 ± 0.06	0.99 ± 0.05	<0.001	0.93 ± 0.06	0.96 ± 0.06	<0.001
BMI (kg/m^2^)	25.7 ± 4.8	25.4 ± 4.8	28.7 ± 4.6	<0.001	24.9 ± 4.6	29.1 ± 4.2	<0.001	24.8 ± 4.5	29.7 ± 4.1	<0.001	25.4 ± 4.9	27.6 ± 4.1	<0.001
DBP (mmHg)	74.5 ± 12.0	74.0 ± 11.7	80.1 ± 12.2	<0.001	72.9 ± 11.3	81.3 ± 12.5	<0.001	73.1 ± 11.3	80.7 ± 12.7	<0.001	74.0 ± 11.9	77.5 ± 12.1	<0.001
SBP (mmHg)	111.4 ± 18.9	110.4 ± 18.0	122.7 ± 21.1	<0.001	108.6 ± 17.1	123.2 ± 20.0	<0.001	108.9 ± 17.1	122.4 ± 20.5	<0.001	110.8 ± 18.5	116.0 ± 18.6	<0.001
PR (bpm)	74.2 ± 10.7	73.9 ± 10.7	77.1 ± 11.1	<0.001	73.5 ± 10.6	77.2 ± 10.7	<0.001	73.5 ± 10.6	77.2 ± 10.8	<0.001	73.9 ± 10.7	76.2 ± 10.9	<0.001
MAP (mmHg)	86.8 ± 13.5	86.1 ± 13.2	94.3 ± 14.1	<0.001	84.8 ± 12.6	95.2 ± 14.1	< 0.001	85.0 ± 12.6	94.6 ± 14.4	<0.001	86.3 ± 13.4	90.4 ± 13.5	<0.001
FBG (mg/dL)	92.9 ± 29.6	89.3 ± 21.0	135.8 ± 62.4	<0.001	88.2 ± 20.6	111.3 ± 48.7	<0.001	89.3 ± 23.4	108.9 ± 44.7	<0.001	91.7 ± 26.9	101.5 ± 43.0	<0.001
TG (mg/dL)	132.2 ± 82.5	127.8 ± 78.2	183.8 ± 109.3	<0.001	115.5 ± 65.8	202.9 ± 105.6	<0.001	118.6 ± 71.3	192.0 ± 99.9	<0.001	109.2 ± 40.3	290.8 ± 117.4	<0.001
Cholesterol (mg/dL)	185.8 ± 39.0	185.2 ± 38.3	192.3 ± 46.6	<0.001	183.1 ± 37.6	197.4 ± 42.5	<0.001	183.0 ± 37.6	197.9 ± 42.4	<0.001	181.2 ± 36.0	217.3 ± 44.0	<0.001
LDL-c (mg/dL)	107.9 ± 32.8	108.0 ± 32.3	106.9 ± 38.5	0.404	106.8 ± 32.0	112.3 ± 36.0	<0.001	106.6 ± 32.0	113.5 ± 35.8	<0.001	107.0 ± 31.4	113.6 ± 40.8	<0.001
HDL-c (mg/dL)	51.4 ± 16.0	51.7 ± 16.1	48.6 ± 15.1	<0.001	53.1 ± 16.4	44.5 ± 12.3	<0.001	52.7 ± 16.4	46.1 ± 13.2	<0.001	52.3 ± 16.0	45.5 ± 14.8	<0.001
WDI scores													
WDI-G *	−0.14 ± 0.20	−0.14 ± 0.20	−0.20 ± 0.19	<0.001	−0.13 ± 0.20	−0.18 ± 0.20	<0.001	−0.13 ± 0.200	−0.18 ± 0.20	<0.001	−0.13 ± 0.200	−0.18 ± 0.21	<0.001
WDI-GC *	0.72 ± 0.40	0.73 ± 0.40	0.60 ± 0.39	<0.001	0.74 ± 0.40	0.65 ± 0.40	<0.001	0.74 ± 0.40	0.64 ± 0.41	<0.001	0.73 ± 0.40	0.64 ± 0.42	<0.001
WDI-GS *	2.84 × 10^−15^ ± 1.00	0.03 ± 1.00	−0.29 ± 0.96	<0.001	0.04 ± 0.99	−0.18 ± 1.00	<0.001	0.04 ± 0.99	−0.19 ± 1.01	<0.001	0.03 ± 0.99	−0.19 ± 1.04	<0.001
WDI-P *	−0.01 ± 0.049	−0.01 ± 0.05	−0.03 ± 0.05	<0.001	−0.01 ± 0.05	−0.02 ± 0.05	<0.001	−0.01 ± 0.05	−0.02 ± 0.047	<0.001	−0.01 ± 0.05	−0.02 ± 0.05	<0.001
WDI-PC *	0.98 ± 0.10	0.98 ± 0.10	0.95 ± 0.10	<0.001	0.98 ± 0.10	0.96 ± 0.10	<0.001	0.98 ± 0.10	0.96 ± 0.10	<0.001	0.98 ± 0.10	0.96 ± 0.10	<0.001
WDI-PS *	7.30 × 10^−16^ ± 1.00	0.03 ± 1.00	−0.32 ± 0.96	<0.001	0.04 ± 1.00	−0.19 ± 0.96	<0.001	0.04 ± 1.00	−0.19 ± 0.97	<0.001	0.02 ± 0.99	−0.14 ± 1.02	<0.001
WDI-I *	−7.58 × 10^−16^ ± 0.35	0.0005 ± 0.35	−0.01 ± 0.35	<0.001	0.0001 ± 0.35	−0.0005 ± 0.35	<0.001	−0.0009 ± 0.35	0.0038 ± 0.35	0.618	0.008 ± 0.35	−0.05 ± 0.36	<0.001
WDI-FG *	−4.42 × 10^−16^ ± 0.26	0.007 ± 0.26	−0.08 ± 0.25	<0.001	0.01 ± 0.26	−0.05 ± 0.24	<0.001	0.01 ± 0.26	−0.05 ± 0.24	<0.001	0.004 ± 0.26	−0.03 ± 0.26	<0.001

* For the detailed calculation methods, please see the Section 2. ^†^ No: without MetS, Yes: having MetS. ** Cluster 1 is labeled as “healthy” and Cluster 2 as “unhealthy” groups; for details, see Supplementary Table S7. The k-means clusters represent data-driven groupings based on metabolic biomarkers and are intended to complement, not replace, clinical MetS definitions. WDI: Westernized diet index, WDI-G: WDI global, WDI-GC: WDI global centralized, WDI-GS: WDI global standardized, WDI-P: WDI population, WDI-PC: WDI population centralized, WDI-PS: WDI population standardized, WDI-I: WDI individual, WDI-FG: WDI food groups, MetS: metabolic syndrome, METs: metabolic equivalents, DBP: diastolic blood pressure, SBP: systolic blood pressure, PR: pulse rate, bpm: beats per minute, FBG: fasting blood glucose, TG: triglycerides, LDL-c: low-density lipoprotein cholesterol, HDL-c: high-density lipoprotein cholesterol, WHO: World Health Organization, ATPIII: Adult Treatment Panel III, IDF: International Diabetes Federation. Overall, these results indicate that the WDI and associated biomarkers were significantly different between individuals with and without MetS, as well as between the healthy and unhealthy K-means clusters, highlighting the associations between a WD and poor metabolic health.

### 3.2. Results from Logistic Regression Models

#### 3.2.1. Crude Logistic Regression Models

The crude associations between various methods of calculating the WDI and the likelihood of having MetS, as estimated by logistic regression models, are shown in Supplementary Table S3. For almost all WDI estimation methods, significant associations were observed with the odds of MetS across the different definitions, with all *p*-values being less than 0.001. The WDI-G and WDI-P demonstrated the strongest associations. Similar trends were observed for the ATPIII and IDF definitions, with significant associations. In contrast, the WDI-I did not show significant associations with MetS, suggesting that individual food component intakes alone were not as strongly associated with MetS compared to the global and population-based methods, as well as the food group-based methods. The WDI-FG demonstrated moderate associations (Supplementary Table S3).

#### 3.2.2. Age and Sex Adjusted Logistic Regression Models

The age and sex-adjusted ORs and 95% CIs for the association between different WDI estimation methods and various definitions of MetS, as assessed through logistic regression models, are presented in Supplementary Table S4. All WDI variants showed significant associations with MetS across the three definitions, with WDI-P exhibiting the strongest associations and other variants (WDI-G, WDI-GC, WDI-GS, WDI-PC, WDI-PS, WDI-I, and WDI-FG) showing similar significant relationships (Supplementary Table S4).

#### 3.2.3. Fully Adjusted Logistic Regression Models

**Figure 1:**
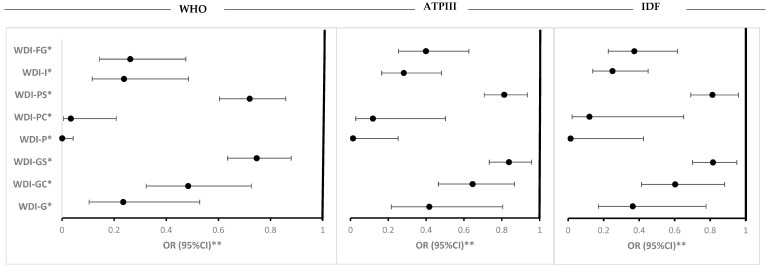
Association (ORs and 95%CIs) between different WDI estimation methods and various MetS definitions in fully adjusted logistic regression models. ** Adjusted for age, sex, METs, current smoking status, and total energy intake. All *p*-values < 0.05. * For the detailed calculation methods, please see the Section 2 (in all methods, higher scores indicate lower adherence to WDPs). WDI: Westernized diet index, WDI-G: WDI global, WDI-GC: WDI global centralized, WDI-GS: WDI global standardized, WDI-P: WDI population, WDI-PC: WDI population centralized, WDI-PS: WDI population standardized, WDI-I: WDI individual, WDI-FG: WDI food groups, OR: odds ratio, CI: confidence interval, MetS: metabolic syndrome, WHO: World Health Organization, ATPIII: Adult Treatment Panel III, IDF: international diabetes federation, METs: metabolic equivalents.

Fully adjusted logistic regression models—adjusted for age, sex, metabolic equivalents (METs), current smoking status, and total energy intake—for assessing the ORs and CIs for the association between different WDI estimation methods and various definitions of MetS are shown in Figure 1 and Table 2.

All WDI estimation methods continued to show significant associations with MetS across all three definitions. The WDI-G demonstrated significant associations with all three MetS definitions (ORs for MetS based on WHO, ATPIII, and IDF were 0.23, 0.42, and 0.36, respectively, *p*-values < 0.001). Similarly, the WDI-GC also showed significant associations with MetS. The ORs ranged from 0.78 for WHO to 0.60 for IDF (*p*-values < 0.001). The WDI-GS demonstrated similar associations with MetS (ORs of 0.75 for WHO, 0.84 for ATPIII, and 0.82 for IDF). Again, all associations were statistically significant (*p*-values < 0.01). The WDI-P showed particularly strong associations with MetS (ORs were 0.001 for WHO, 0.014 for ATPIII, and 0.014 for IDF, *p*-values < 0.014) (Figure 1 and Table 2).

The WDI-PC and WDI-PS also demonstrated significant associations with MetS (ORs for the WDI-PC were 0.03 for the WHO, 0.12 for the ATPIII, and 0.12 for the IDF, *p*-values < 0.014). The WDI-PS showed ORs of 0.72 for WHO, 0.81 for ATPIII, and 0.81 for IDF (*p*-values < 0.014). The WDI-I showed significant associations with MetS across all three definitions (ORs ranging from 0.24 for the WHO definition to 0.25 for the IDF definition, *p*-values < 0.001). Finally, the WDI-FG method showed significant associations with MetS (ORs of 0.26 for WHO, 0.40 for ATPIII, and 0.37 for IDF (*p* <0.001 for all)) (Figure 1 and Table 2).

### 3.3. Results from Linear Regression Models

#### 3.3.1. Crude Linear Regression Models

The crude linear regression models investigating the associations between various WDI methods and a range of metabolic biomarkers are shown in Supplementary Table S5. Significant inverse associations were observed between the WDI and several metabolic biomarkers. The WDI-G, WDI-P, and WDI-FG exhibited strong negative associations (in all methods, higher scores indicate lower adherence to WDPs) with WC, HC, and BMI. In addition, significant inverse associations were observed across all WDI methods for the blood pressure-related biomarkers, including SBP and DBP. The strongest associations were found for SBP, DBP, and MAP with the WDI-P (all *p* < 0.001). In addition, the WDI-P method showed the most robust associations with TG, FBG, and cholesterol (all *p* < 0.001). Interestingly, no significant associations were found between LDL-c and HDL-c levels across most WDI methods, except for a positive, significant association with HDL-c in WDI-I (*p* < 0.001) (Supplementary Table S5).

#### 3.3.2. Age and Sex Adjusted Linear Regression Models

Supplementary Table S6 presents the associations between various methods of estimating the WDI and different metabolic biomarkers in linear regression models adjusted for age and sex. All WDI methods (global, population-based, individual, and food groups) demonstrated significant associations (*p* < 0.001) with multiple biomarkers. Blood pressure measurements, including DBP, SBP, and MAP, were significantly associated with all WDI estimation methods. The strongest associations were observed for SBP and DBP with the WDI-G and WDI-P (*p*-values < 0.001). FBG exhibited strong associations, particularly with the WDI-G and WDI-P, with all *p*-values < 0.001. Similarly, TG and cholesterol showed significant associations with most WDI methods, with the WDI-G showing the most pronounced effects. In contrast, associations with LDL-c were weaker and not consistently significant across all WDI methods. For HDL-c, the most significant and positive associations were observed with the WDI-P, WDI-PC, and WDI-I, all with *p*-values < 0.05 (Supplementary Table S6).

#### 3.3.3. Fully Adjusted Linear Regression Models

**Figure 2:**
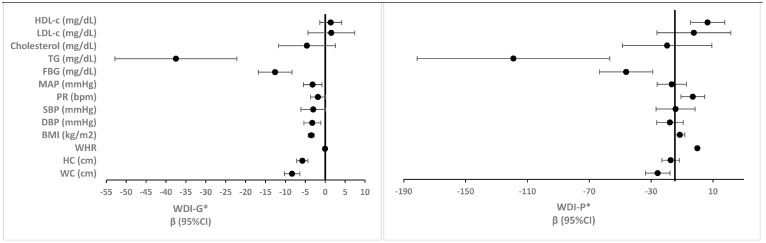

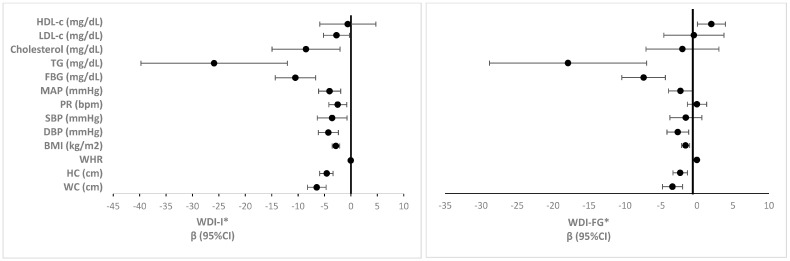
Association (β and 95%CIs) between different WDI estimation methods * and various metabolic (bio)markers in fully adjusted linear regression models. * Adjusted for age, sex, METs, current smoking status, and total energy intake. For detailed calculation methods, please refer to Section 2. WDI: Westernized diet index, β: beta, CI: confidence interval, MetS: metabolic syndrome, DBP: diastolic blood pressure, SBP: systolic blood pressure, PR: pulse rate, bpm: beats per minute, FBG: fasting blood glucose, TG: triglycerides, Choles: cholesterol, LDL-c: low-density lipoprotein-cholesterol, HDL-c: high-density lipoprotein-cholesterol, ARS: adjusted R-squared, METs: metabolic equivalents.

Fully adjusted linear regression models—adjusted for age, sex, METs, smoking status, and total energy intake—investigating the associations between different WDI methods (higher scores indicate lower adherence to WDPs) and various metabolic biomarkers are presented in Figure 2 and Table 3. For DBP, all methods revealed significant associations, with the WDI-G and WDI-P showing strong associations (β = −3.24 and β = −17.88, respectively); this corresponds to a 10% increase in WDI-G (range: –2.03 to 1.11; 10% = 0.31 units) and WDI-P (range: –0.36 to 0.42; 10% = 0.08 units) was associated with decreases of approximately 1.02 mmHg and 1.39 mmHg in DBP, respectively. SBP was associated with several WDI methods, particularly the WDI-P (β = −14.20) and WDI-PS (β = −7.10); in other words, a 10% increase in WDI-P (10% = 0.08 units) and WDI-PS (10% = 1.60 units) was associated with decreases of approximately 1.11 mmHg and 11.38 mmHg in SBP, respectively. MAP was negatively related to all WDI methods, e.g., WDI-G (β = −3.165) and WDI-P (β = −16.65); expressed differently, a 10% increase in WDI-G (10% = 0.31 units) and WDI-P (10% = 0.08 units) was associated with decreases of approximately 0.99 mmHg and 1.30 mmHg in MAP, respectively. FBG exhibited strong negative associations across all WDI methods, particularly with the WDI-G (β = −12.59) and WDI-P (β = −46.16); in other words, a 10% increase in WDI-G (10% = 0.31 units) and WDI-P (10% = 0.08 units) was associated with decreases of approximately 3.95 mg/dL and 3.60 mg/dL in FBG, respectively. TG was negatively associated with all methods, with the WDI-G and WDI-P showing strong associations (β = −37.502 and β = −119.093, respectively); in other words, a 10% increase in WDI-G (10% = 0.31 units) and WDI-P (10% = 0.08 units) was associated with decreases of approximately 11.78 mg/dL and 9.29 mg/dL in TG, respectively. Cholesterol, LDL-c, and HDL-c did not show significant associations with most WDI methods, except for LDL-c and cholesterol with the WDI-I (β = −2.73 and β = −8.51, respectively); expressed differently, a 10% increase in WDI-I (range: –3.59 to 1.07; 10% = 0.47 units), was associated with decreases of approximately 1.27 mg/dL and 3.97 mg/dL in LDL-C and total cholesterol, respectively. HDL-c exhibited a significant positive association with the WDI-FG (β = 2.00); in other words, a 10% increase in WDI-FG (range: –1.91 to 2.81; 10% = 0.47 units) (higher scores indicate lower adherence to WDPs) was associated with an increase of approximately 0.94 mg/dL in HDL-c, but no significant associations were observed for the other WDI methods (Figure 2 and Table 3). Given the large sample size, statistical significance was interpreted in conjunction with the magnitude of the effect size. Although several associations reached high statistical significance, their biological relevance was evaluated based on the direction and magnitude of the estimated effects.

Taken together, all WDI estimation methods remained significantly associated with MetS in the fully adjusted logistic regression models, with the population-based and food group methods showing robust associations (Figure 1 and Table 2). In addition, the results from linear regression models suggest that several WDI methods were consistently associated with various metabolic biomarkers, with the strongest effects generally observed for the global and population-based WDI methods (Figure 2 and Table 3).

### 3.4. Results from ROC Curves

**Figure 3:**
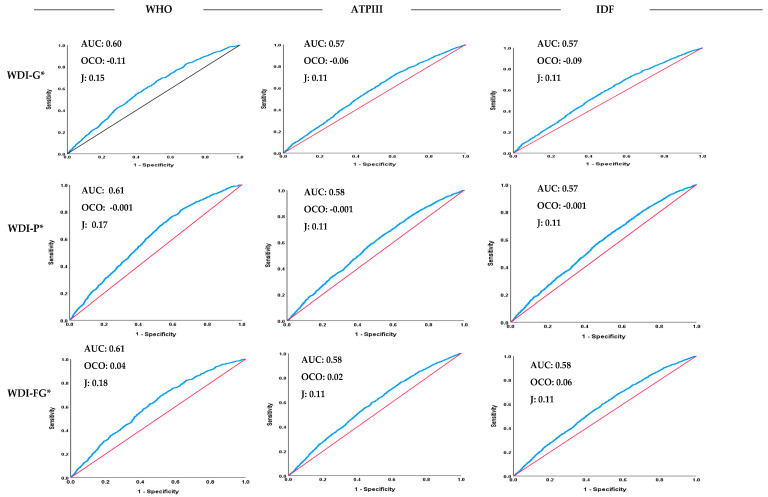
Receiver Operating Characteristic (ROC) curves for WDI in predicting metabolic syndrome (MetS) based on different definitions. For all Null hypotheses: true area = 0.5. AUC: area under the curve, WDI: Westernized Diet Index, WHO: World Health Organization, ATPIII: Adult Treatment Panel III, IDF: International Diabetes Federation, OCO: optimal cutoff (threshold), J: Youden’s index = sensitivity + Specificity − 1. * For the details, please see Section 2.

The performance of the WDI in predicting MetS is presented in Figure 3. The AUC values for WDI varied across the different definitions. Overall, the WDI demonstrated moderate performance in predicting MetS across the various definitions, with similar AUC values observed for the global, population, and food group-based WDI models (Figure 3).

### 3.5. Results from K-Means Clustering

The final cluster centers for health indicators and the WDI for the healthy (Cluster 1) and unhealthy (Cluster 2) clusters are presented in Supplementary Table S7. The unhealthy cluster showed higher levels of key biomarkers, including WC, DBP, SBP, FBG, TG, cholesterol, and LDL-c, compared with the healthy cluster. In contrast, HDL-c was higher in the healthy cluster. The WDI values were slightly less negative in the healthy cluster, indicating a milder WD pattern than in the unhealthy cluster. When comparing K-means clusters (Table 1), Cluster 2 (unhealthy) exhibited significantly lower WDI values (indicating higher adherence to WDPs) across all subcategories (WDI global, population, individual, and food groups) compared to Cluster 1 (healthy), with *p*-values consistently below 0.001. This trend was also observed in WDI-P measures, where Cluster 2 showed lower mean values (e.g., WDI-P: −0.018 in Cluster 2 vs. −0.010 in Cluster 1). Additionally, Cluster 2 showed higher biomarker levels than Cluster 1. For instance, TG levels in Cluster 2 were significantly elevated (291 mg/dL) compared to Cluster 1 (109 mg/dL) (Table 1). LDL-c levels did not show significant differences across MetS categories. However, significant differences were found between K-means clusters, with Cluster 2 displaying higher LDL-c levels compared to Cluster 1 (*p* < 0.001) (Table 1). The concordance between lower WDI scores and the metabolically ‘unhealthy’ cluster provides additional, definition-independent support for the construct validity of the WDI.

**Table 3:** Association (β and 95%CIs) between different WDI estimation methods * and various metabolic (bio)markers in fully adjusted linear regression models **.

Bio (Marker)	WDI-G	R/ARS	*p*-Value	WDI-GC	R/ARS	*p*-Value	WDI-GS	R/ARS	*p*-Value	WDI-P	R/ARS	*p*-Value	WDI-PC	R/ARS	*p*-Value	WDI-PS	R/ARS	*p*-Value	WDI-I	R/ARS	*p*-Value	WDI-FG	R/ARS	*p*-Value
WC (cm)	−8.33 (−10.28, −6.38)	0.39/0.15	<0.001	−4.16 (−5.14, −3.19)	0.39/0.15	<0.001	−1.68 (−2.07, −1.28)	0.39/0.15	<0.001	−25.77 (−33.73, −17.82)	0.38/0.14	<0.001	−12.89 (−16.86, −8.91)	0.38/0.14	<0.001	−1.25 (−1.64, −0.87)	0.38/0.14	<0.001	−6.47 (−8.23, −4.70)	0.38/0.14	<0.001	−3.40 (−4.80, −2.01)	0.37/0.14	<0.001
HC (cm)	−5.74 (−7.14, −4.34)	0.32/0.10	<0.001	−2.87 (−3.57, −2.57)	0.32/0.10	<0.001	−1.15 (−1.44, −0.87)	0.32/0.10	<0.001	−17.35 (−23.08, −11.63)	0.31/0.09	<0.001	−8.68 (−11.54, −5.81)	0.31/0.09	<0.001	−0.84 (−1.12, −0.57)	0.31/0.09	<0.001	−4.57 (−5.93, −3.39)	0.32/0.10	<0.001	−2.34 (−3.34, −1.33)	0.30/0.09	<0.001
WHR	−0.03 (−0.04, −0.02)	0.42/0.17	<0.001	−0.01 (−0.02, −0.01)	0.42/0.174	<0.001	−0.006 (−0.01, −0.004)	0.42/0.17	<0.001	−0.10 (−0.14, −0.05)	0.41/0.17	<0.001	−0.05 (−0.07, −0.03)	0.41/0.17	<0.001	−0.005 (−0.007, −0.003)	0.41/0.17	<0.001	−0.02 (−0.03, −0.01)	0.41/0.17	<0.001	−0.01 (−0.02, −0.005)	0.41, 0.17	0.002
BMI (kg/m^2^)	−3.52 (−4.30, −2.74)	0.38/0.14	<0.001	−1.76 (−2.15, −1.37)	0.38/0.14	<0.001	−0.71 (−0.86, −0.55)	0.38/0.14	<0.001	−11.44 (−14.64, −8.25)	0.36/0.13	<0.001	−5.72 (−7.32, −4.32)	0.36/0.13	<0.001	−0.56 (−0.71, −0.40)	0.36/0.130	<0.001	−2.84 (−3.55, −2.14)	0.37/0.13	<0.001	−1.59 (−2.15, −1.03)	0.35/0.12	<0.001
DBP (mmHg)	−3.24 (−5.36, −1.14)	0.29/0.08	0.003	−1.62 (−2.68, −0.56)	0.29/0.08	0.003	−0.65 (−1.08, −0.23)	0.29/0.08	0.003	−17.88 (−26.45, −9.31)	0.29/0.08	<0.001	−8.94 (−13.23, −4.65)	0.29/0.08	<0.001	−0.87 (−1.287, −0.453)	0.29/0.08	<0.001	−4.27 (−6.18, −2.37)	0.29/0.08	<0.001	−2.67 (−4.18, −1.17)	0.29/0.08	<0.001
SBP (mmHg)	−3.01 (−6.14, 0.11)	0.40/0.16	0.059	−1.50 (−3.06, 0.05)	0.40/0.16	0.0659	−0.61 (−1.23, 0.02)	0.40/0.16	0.059	−14.20 (−26.86, −1.54)	0.40/0.16	0.028	−7.10 (−13.43, −0.77)	0.40/0.16	0.028	−0.69 (−1.31, −0.07)	0.40/0.16	0.028	−3.56 (−6.38, −0.74)	0.40/0.16	0.014	−1.55 (−3.77, 0.67)	0.40/0.16	0.171
PR (bpm)	−1.83 (−3.71, 0.05)	0.20/0.04	0.056	−0.91 (−1.85, 0.02)	0.20/0.04	0.056	−0.37 (−0.75, 0.01)	0.20/0.04	0.056	−3.08 (−10.71, 4.56)	0.20/0.04	0.430	−1.54 (−5.36, 2.28)	0.20/0.04	0.430	−0.15 (−0.52, 0.22)	0.20/0.04	0.430	−2.5 (−4.19, −0.80)	0.21/0.04	0.004	−0.01 (−1.35, 1.33)	0.20/0.04	0.989
MAP (mmHg)	−3.16 (−5.49, −0.84)	0.35/0.12	0.008	−1.58 (−2.75, −0.42)	0.35/0.12	0.008	−0.64 (−1.10, −0.17)	0.35/0.12	0.008	−16.65 (−26.10, −7.21)	0.35/0.12	0.001	−8.33 (−13.05, −3.60)	0.35/0.12	0.001	−0.81 (−1.27, −0.35)	0.35/0.12	0.001	−4.04 (−6.14, −1.93)	0.35/0.12	<0.001	−2.30 (−3.95, −0.64)	0.35/0.12	0.007
FBG (mg/dL)	−12.59 (−16.82, −8.36)	0.24/0.05	<0.001	−6.29 (−8.41, −4.18)	0.24/0.05	<0.001	−2.53 (−3.38, −1.68)	0.24/0.05	<0.001	−46.17 (−63.36, −28.97)	0.23/0.05	<0.001	−23.08 (−31.68, −14.48)	0.23/0.05	<0.001	−2.25 (−3.08, −1.41)	0.23/0.05	<0.001	−10.51 (−14.34, −6.68)	0.23/0.05	<0.001	−7.41 (−10.46, −4.40)	0.23/0.05	<0.001
TG (mg/dL)	−37.50 (−52.81, −22.19)	0.16/0.02	<0.001	−18.75 (−26.41, −11.10)	0.16/0.02	<0.001	−7.55 (−10.63, −4.47)	0.16/0.02	<0.001	−119.09 (−181.37, −56.81)	0.15/0.02	<0.001	−59.55 (−90.69, −28.41)	0.15/0.02	<0.001	−5.79 (−8.83, −2.76)	0.15/0.02	<0.001	−25.89 (−39.75, −12.03)	0.14/0.02	<0.001	−17.92 (−28.84, −7.00)	0.14/0.02	0.001
Cholesterol (mg/dL)	−4.6 (−11.73, 2.53)	0.17/0.02	0.206	−2.30 (−5.86, 1.26)	0.17/0.02	0.206	−0.93 (−2.36, 0.51)	0.17/0.02	0.206	−19.68 (−48.62, 9.26)	0.17/0.02	0.183	−9.84 (−24.31, 4.63)	0.17/0.02	0.183	−0.96 (−2.37, 0.45)	0.17/0.02	0.183	−8.51 (−14.94, −2.07)	0.17/0.03	0.010	−2.02 (−7.10, 3.05)	0.16/0.02	0.434
LDL-c (mg/dL)	1.53 (−4.34, 7.40)	0.11/0.01	0.610	0.76 (−2.17, 3.70)	0.11/0.01	0.610	0.31 (−0.87, 1.49)	0.11/0.01	0.610	−2.37 (−26.22, 21.48)	0.11/0.01	0.845	−1.19 (−13.11, 10.74)	0.11/0.01	0.845	−0.11 (−1.28, 1.04)	0.11/0.01	0.845	−2.73 (−5.20, −0.27)	0.29/0.08	0.029	−0.43 (−4.61, 3.74)	0.11/0.01	0.838
HDL-c (mg/dL)	1.37 (−1.35, 4.10)	0.29/0.08	0.323	0.69 (−0.68, 2.05)	0.29/0.08	0.323	0.28 (−0.27, 0.82)	0.29/0.08	0.323	6.51 (−4.56, 17.59)	0.29/0.08	0.249	3.26 (−2.28, 8.79)	0.29/0.08	0.249	0.32 (−0.22, 0.86)	0.29/0.08	0.249	−0.59 (−5.90, 4.72)	0.11/0.01	0.828	2.00 (0.06, 3.93)	0.29/0.08	0.044

** Adjusted for age, sex, METs, current smoking status, and total energy intake. * For the detailed calculation methods, please see Section 2 (in all methods, higher scores indicate lower adherence to WDPs). WDI: Westernized diet index, WDI-G: WDI global, WDI-GC: WDI global centralized, WDI-GS: WDI global standardized, WDI-P: WDI population, WDI-PC: WDI population centralized, WDI-PS: WDI population standardized, WDI-I: WDI individuald, WDI-FG: WDI food groupse, β: beta, CI: confidence interval, MetS: metabolic syndrome, DBP: diastolic blood pressure, SBP: systolic blood pressure, PR: pulse rate, bpm: beats per minute, FBG: fasting blood glucose, TG: triglycerides, LDL-c: low-density lipoprotein cholesterol, HDL-c: high-density lipoprotein cholesterol, R: R-squared, ARS: adjusted R-squared, METs: metabolic equivalents.

## 4. Discussion

This validation study demonstrated strong and significant associations between adherence to the WD, as assessed by a novel dietary index, the WDI, considering 30 food groups/dietary constituents, and metabolic health outcomes. Across all three MetS definitions (WHO, ATPIII, IDF), higher WDI scores (indicating lower adherence to WD) were consistently associated with lower odds of MetS. Among the various scoring methods, the WDI-G and the WDI-I reduced the odds of MetS (WHO definition), a major metabolic complication with a global prevalence of over 25% [4], by up to 99%. Higher WDI scores (indicating lower adherence to WD) were also significantly associated with more favorable metabolic biomarker profiles. The WDI-P method demonstrated the most pronounced inverse associations with metabolic markers, i.e., a 25.7 cm decrease in WC, a 46 mg/dL reduction in FBG, and a 14.2 mmHg drop in SBP per unit increase of the WDI; in other words, a 10% increase in WDI-P (range: –0.36 to 0.42; 10% = 0.08 units) was associated with decreases of approximately 2.00 cm in WC, 3.59 mg/dL in FBG, and 1.11 mmHg in SBP. WDI-FG and WDI-I showed moderate associations with MetS and biomarkers. As expected in large population-based studies, even small effect sizes may achieve statistical significance; therefore, the findings should be interpreted with an emphasis on effect magnitude and potential clinical relevance rather than *p*-values alone.

Additionally, ROC analyses indicated that WDI-P and WDI-FG had the highest discriminatory power for MetS (AUC of 0.61), slightly outperforming WDI-G. The findings suggest, for the first time, that while all WDI scoring methods modestly capture the metabolic impact of a WD, population-based and standardized approaches provided the strongest discriminatory value for metabolic risk. According to established benchmarks, the observed AUC and Youden index values reflected modest diagnostic accuracy; however, they still suggest discrimination beyond chance and should be interpreted within a construct validation rather than a diagnostic framework.

The WDI is inherently adaptable to diverse dietary contexts, making it suitable for use across global populations with varying food cultures. Because the index is based on food groups/items and standardized scoring rather than fixed dietary prescriptions, it can accommodate plant-based diets and mixed dietary patterns common in Asian, African, and other non-Western settings, as well as in Western settings, by recalibrating population-based reference distributions while preserving the underlying Westernization construct. In addition, the WDI is well-suited for integration within digital health tools, including dietary assessment apps and AI-assisted monitoring platforms, enabling scalable, real-time evaluation of dietary patterns in both research and applied settings. From a clinical perspective, the WDI may serve as a practical tool for monitoring dietary risk in individuals at elevated risk of MetS, supporting targeted lifestyle interventions and longitudinal follow-up in preventive and clinical care.

Our findings align with previous studies examining the association between WD adherence and metabolic health. For instance, a population-based prospective cohort study (the Malmö Diet and Cancer Study (MDCS)) [32] found that higher WD adherence was associated with adverse cardiometabolic traits at baseline and at follow-up after ~16 years, as well as an increased risk of MetS. The strong inverse associations we observed between the WDI and MetS are consistent with the finding that individuals with higher intakes of processed foods, sugars, and unhealthy fats tend to exhibit worse metabolic health markers [32]. In accordance, a systematic review/meta-analysis [33] concluded that “Healthy” and “Meat/Western” dietary patterns are significantly associated with reduced and increased MetS risk, respectively.

Furthermore, our study supports previous findings [9,32,33,34] that dietary patterns associated with the WD are associated with key biomarkers of MetS, including WC, BMI, BP, and glucose levels, as observed in the present study. Our results also corroborate studies [9,34,35,36,37,38,39] showing that food groups characteristic of a WD (e.g., red meat, sugary beverages, refined grains) are associated with markers of adiposity, insulin resistance, and lipid profiles, among others. Additionally, the ROC curve analysis in our study, indicating a moderate discriminatory ability of the WDI for MetS, aligns with findings from similar studies that predict MetS using dietary patterns [40]. Although our AUC values were moderate (0.57 to 0.61), they still highlight the potential of the WDI as a tool for identifying individuals at higher risk of MetS.

When comparing the WDI with other diet quality indices, several distinguishing but also similar aspects emerge. The Alternative Healthy Eating Index (AHEI) [7,41], for instance, a commonly used, validated index in population-based studies, assesses adherence to the Dietary Guidelines for Americans, promoting fruits, vegetables, whole grains, and lean proteins while reducing unhealthy fats, sugars, and sodium. In contrast, the WDI explicitly captures the extent to which an individual adheres to an undesired but arguably more typical WDPs, characterized by a high intake of processed foods, red meats, sugary beverages, and refined grains. This makes the WDI more specific in identifying metabolic risks associated with poor dietary choices, while the AHEI focuses on adherence to national dietary guidelines aimed at improving general health [15].

The MDS [7,16,19], a reliable and valid index that evaluates adherence to the MD, rich in fruits, vegetables, legumes, whole grains, fish, and healthy fats, can be viewed as the opposite of the WDI. However, the MDS [16,19] is only a food-group-based index, whereas the WDI is more comprehensive, encompassing not only food groups but also specific nutrients (e.g., fats, sugars, and fiber), non-nutrients (e.g., phytochemicals), and processed food consumption. This broader scope of the WDI allows capturing a wider range of dietary patterns and behaviors, allowing a nuanced assessment of various dimensions of dietary habits and their association with metabolic health, whereas the MDS primarily assesses adherence to an MD pattern and may not fully account for the complex interactions between different nutrients and other dietary elements that contribute to metabolic outcomes.

The DQI-I [42] also differs from the WDI as it evaluates dietary diversity and nutrient adequacy. The DQI emphasizes a holistic assessment of diet quality by considering both beneficial and detrimental dietary components, such as the balance between fruits, vegetables, and nutrient-dense foods vs. consuming unhealthy fats and sugars [42]. Additionally, the WDI includes additional detrimental components, such as high intake of processed foods and refined grains, as well as beneficial components, such as phytochemicals, vitamins, and minerals, all of which have been linked to improved metabolic health outcomes. While the DQI-I provides a more general measure of diet quality, the WDI’s targeted evaluation offers a more direct and practical measure of the dietary influences on metabolic health, making it particularly useful in identifying individuals at higher risk for MetS and related conditions.

Unlike the WDI, the DASH (Dietary Approaches to Stop Hypertension) diet score [43] focuses on a single health indicator: reducing high blood pressure through a diet rich in fruits, vegetables, whole grains, lean proteins, and low-fat dairy, while emphasizing low sodium intake. This index has been shown to improve heart health and MetS and reduce hypertension [43,44]. While the DASH diet focuses on increasing beneficial nutrient-dense foods, it does not explicitly highlight the detrimental effects of a WD pattern, such as the overconsumption of processed and refined food products. Through its literature-based relationship with several cardiometabolic markers, the WDI provides a more robust discriminatory and predictive power for several chronic conditions, including obesity, insulin resistance, and MetS.

Despite the valuable insights our study provides, several limitations warrant consideration. First, our study’s cross-sectional design limits the ability to establish causal relationships between WD adherence and metabolic outcomes. While strong associations were observed, the directionality of these relationships remains not fully established, and future prospective studies are needed to confirm causality. Second, the use of self-reported dietary intake data introduces the potential for recall bias and misreporting. Although we attempted to mitigate these concerns through a validated FFQ, measurement error remains a concern when relying on self-reported data.

Additionally, while the WDI provides a useful measure of dietary patterns, it is inherently limited by its reliance on the availability of specific food items and nutrient information, which may not fully capture the dietary nuances of individuals from very diverse cultural or geographical backgrounds. Furthermore, the generalizability of our findings may be limited, as our sample population may not fully represent all demographic groups, and the results may not be applicable to populations with different dietary patterns or risk profiles. Although the WDI captures WDPs beyond meat consumption alone and integrates multiple food groups beyond animal products, the heterogeneity of vegetarian and plant-based diets, including highly processed plant-based foods, may influence index performance; however, due to sample size limitations, subgroup analyses by dietary patterns (e.g., vegetarian vs. omnivorous) were not feasible in the current study, underscoring the need for further validation in populations where such dietary patterns are prevalent or rapidly evolving. Although models were adjusted for key demographic and lifestyle factors, the limited availability of data on additional potential confounders, such as more detailed information on the socioeconomic status and medication use, among others, raises the possibility of residual confounding, which should be addressed in future studies with more comprehensive covariate information. In addition, as the validation was limited to an Iranian cohort, the global applicability of the WDI should be interpreted cautiously, and future studies in diverse cultural settings, including populations with prevalent vegetarian or plant-based diets undergoing dietary transition, are needed to confirm its broader relevance. Lastly, while the metabolic biomarkers used in our analysis are important indicators of metabolic health, they do not capture the full spectrum of metabolic dysfunction, including genetic predispositions and other unmeasured factors that may influence the association between diet and health. Future studies should incorporate more comprehensive biomarkers and longitudinal designs to understand the long-term effects of WD patterns on metabolic health.

Our study boasts several key strengths that significantly enhance its value in the field of nutrition and metabolic health research. First, the application of the WDI enabled a comprehensive, multidimensional assessment of dietary patterns, considering not only food groups but also nutrients and non-nutrient aspects of the diet. This multifaceted approach provides a more holistic perspective on the impact of diet on metabolic health compared to single-component indices. Second, the study’s design used a large, diverse sample, enhancing the external validity and generalizability of our findings to a broader population. Additionally, by incorporating multiple diagnostic definitions of MetS (WHO, ATPIII, IDF), our study provides a robust and comprehensive evaluation of metabolic health across various criteria, thereby strengthening our conclusions. The use of advanced statistical techniques, including linear mixed models and unsupervised k-means clustering, allowed us to account for potential confounders and individual variability, thereby providing more reliable estimates of the associations between WDI scores and metabolic biomarkers. Moreover, the inclusion of both global and population-based WDI scoring methods enabled a nuanced comparison, highlighting the WDI’s discriminatory and predictive value across contexts and enhancing the precision of our results. The comprehensive nature of this approach reduces measurement bias and strengthens the reliability of the findings. Our study also stands out due to the inclusion of a wide range of metabolic biomarkers, providing a more in-depth understanding of the associations between diet and metabolic health outcomes. Finally, our findings contribute novel insights to the existing literature by not only validating the WDI as a predictor of metabolic risk but also identifying specific methods within the WDI, e.g., WDI-G, WDI-P, and WDI-FG, that are most strongly associated with adverse health outcomes.

The WDI offers practical utility for both population-based and clinical research. In epidemiological studies, the WDI enables standardized monitoring of dietary Westernization across populations and over time, facilitating comparisons between regions and supporting surveillance of nutrition transition and metabolic risk. In clinical and preventive settings, the WDI may be used to identify individuals or subgroups with high adherence to WDPs and to monitor dietary changes. It is important to emphasize that the WDI variants evaluated in this study were developed as dietary exposure metrics for epidemiological and clinical research, not as clinical screening or diagnostic tools. Consequently, their performance should not be judged solely by clinical criteria, but rather by their ability to capture meaningful variations in dietary patterns associated with metabolic health at the population level. Future research should focus on validating the WDI in culturally diverse populations with varying dietary traditions, including predominantly plant-based or mixed dietary patterns, and on integrating the index into digital and e-health platforms (e.g., dietary apps and AI-assisted monitoring systems) to enhance scalability, real-time assessment, and personalized nutrition interventions.

## 5. Conclusions

This validation study demonstrates that the WDI is an effective nutritional tool for assessing dietary patterns. By capturing both food group and (non-)nutrient-related aspects of the WD, both health-promoting and health-detrimental ones, the WDI provides a comprehensive measure of dietary adherence that is linked to key metabolic markers such as WC, FBG, and blood pressure. Our findings validate the WDI as a modest predictor of MetS, reinforcing its potential for use in clinical settings and public health applications. Overall, the findings support the use of WDI variants as valid tools for characterizing WDPs in research settings, while highlighting that their application should remain distinct from clinical diagnosis. These results underscore the importance of considering dietary patterns, particularly those characteristic of the WD, in the prevention and management of metabolic disorders. The high consistency of our results with international studies underlines the generalizability of the WDI across different populations. This is particularly important given the global spread of WD habits and their associated metabolic risks. Future research should aim to refine the WDI further and investigate its potential causal role in the development of metabolic conditions, using longitudinal cohorts and dietary intervention studies, thereby advancing its application as a valid nutritional tool for monitoring dietary transitions and patterns and for guiding strategies for NCD prevention.

## Figures and Tables

**Table 2:** Association (ORs and 95%CIs) between different WDI estimation methods and various MetS definitions in fully adjusted logistic regression models *.

Calculation Methods **	WHO	*p*-Value	ATPIII	*p*-Value	IDF	*p*-Value
WDI-G	0.23 (0.10, 0.53)	<0.001	0.42 (0.22, 0.80)	0.009	0.36 (0.17, 0.78)	0.009
WDI-GC	0.48 (0.32, 0.73)	<0.001	0.65 (0.46, 0.87)	0.009	0.60 (0.41, 0.88)	0.009
WDI-GS	0.75 (0.63, 0.88)	<0.001	0.84 (0.73, 0.96)	0.009	0.82 (0.70, 0.95)	0.009
WDI-P	0.001 (0.000032, 0.043)	<0.001	0.01 (0.001, 0.25)	0.004	0.01 (0.000483, 0.42)	0.014
WDI-PC	0.03 (0.006, 0.21)	<0.001	0.12 (0.03, 0.50)	0.004	0.12 (0.02, 0.65)	0.014
WDI-PS	0.72 (0.60, 0.86)	<0.001	0.81 (0.71, 0.93)	0.004	0.81 (0.69, 0.96)	0.014
WDI-I	0.24 (0.12, 0.48)	<0.001	0.28 (0.16, 0.48)	<0.001	0.25 (0.14, 0.45)	<0.001
WDI-FG	0.26 (0.14, 0.47)	<0.001	0.40 (0.25, 0.63)	<0.001	0.373 (0.23, 0.62)	<0.001

* Adjusted for age, sex, METs, current smoking status, and total energy intake. ** For detailed calculation methods, please refer to the Section 2 (in all methods, higher scores indicate lower adherence to WDPs). WDI: Westernized diet index, WDI-G: WDI global, WDI-GC: WDI global centralized, WDI-GS: WDI global standardized, WDI-P: WDI population, WDI-PC: WDI population centralized, WDI-PS: WDI population standardized, WDI-I: WDI individual, WDI-FG: WDI food groups, OR: odds ratio, CI: confidence interval, MetS: metabolic syndrome, WHO: World Health Organization, ATPIII: Adult Treatment Panel III, IDF: International Diabetes Federation, METs: metabolic equivalents.

## Data Availability

The data supporting the findings of this study are available from the FASA cohort consortium; however, restrictions apply to the availability of these data, which were used in the current study. Data are available from the consortium upon reasonable request.

## Supplementary Materials

The following supporting information can be downloaded at: https://www.mdpi.com/article/10.3390/nu18020349/s1, Figure S1: Flow diagram of participants through the study; Figure S2: Decision Guide for WDI: Illustrating When and Why to Select Different WDI Calculation Methods; Table S1: Cutoff values and components used to classify participants according to MetS definitions; Table S2: Global daily mean intake of food parameters (± SD) and their corresponding Westernized Diet Index (WDI) coefficients; Table S3: Association (ORs and 95%CIs) between different WDI estimation methods and various MetS definitions in crude logistic regression models; Table S4: Association (ORs and 95%CIs) between different WDI estimation methods and various MetS definitions in logistic regression models adjusted for age and sex; Table S5: Association (β and 95%CIs) between different WDI estimation methods* and various metabolic (bio)markers in crude linear regression models; Table S6: Association (β and 95%CIs) between different WDI estimation methods* and various metabolic (bio)markers in linear regression models adjusted for age and sex; Table S7: Final cluster centers for health indicators and WDI, showing distinct patterns in healthy and unhealthy clusters [22,24,26,28,31,45,46,47,48,49,50,51,52,53,54,55,56,57,58,59].
