# Supplementary material for: A Global Nutritional Tool for Monitoring Westernized Dietary Transition: Validation of the Westernized Diet Index Using a Large Population Sample and Biomarkers of Metabolic Health
Source: Nutrients. 2026 Jan 21;18(2):349. doi: 10.3390/nu18020349 (PMC12845090; doi:10.3390/nu18020349)
Supplement: Supplementary file 1 [file nutrients-18-00349-s001.zip › nutrients-4080392-supplementary.pdf]

**Supplementary Table S1.** Cutoff values and components used to classify participants according to MetS definitions.

| Components | WHO [1] | NCEP: ATPIII [2,3] | IDF [4] |
|---|---|---|---|
| Mandatory component(s) | GI, IGT or DM, and/or IR together with two or more of the following components: | Three or more of the following components: | Central obesity as defined by ethnic/racial, specific WC, and two of the following components: |
| Blood pressure | ≥ 140/90 mmHg | ≥130/85 mmHg | ≥ 130/85 mmHg |
| Triglycerides | ≥ 150 mg/dl and/or low HDL-c: Men < 35 mg/dl; Female < 39 mg/dl | ≥150 mg/dl | ≥ 150 mg/dl |
| Central obesity | Men: WHR > 90 cm; Women: WHR > 85 cm And/or BMI > 30 $kg/m^2$ | Men: WC > 101 cm; Women: > 88.9 cm | - |
| FBG | - | ≥ 110 mg/dl | FBG ≥100 mg/dl |
| HDL-c | - | Men: < 40 mg/dl; Female: < 50 mg/dl | Men: <40 mg/dl; Women: <50 mg/dl |

MetS: metabolic syndrome; GI: glucose intolerance; IGT: impaired glucose tolerance; DM: diabetes mellitus; IR: insulin resistance; WHR: waist to hip ratio; WC: waist circumference; HT: hypertension; FBG: fasting blood glucose; HDL: High-density lipoprotein, WHO: World Health Organization, NCEP: ATPIII: National Cholesterol Education Program's Adult Treatment Panel III, IDF: International Diabetes Federation.

**Supplementary Table S2**. Global daily mean intake of food parameters (± SD) and their corresponding Westernized Diet Index (WDI) coefficients.

| Food parameter | Global daily mean intake (unit/day) ± SD* | WDI Coefficients [5] | Fractional Rank of the WDI Coefficients |
|---|---|---|---|
| Energy (kcal) | 2056 ± 338 | -0.2395 | 24.14 |
| Carbohydrate (g) | 272.2 ± 40.0 | 0.0584 | 48.28 |
| Protein (g) | 79.4 ± 13.9 | 0.0829 | 51.72 |
| Total fat (g) | 71.4 ± 19.4 | -0.0507 | 44.83 |
| Healthy fatty acids | | | |
| MUFA$ (g) | 27.0 ± 6.1 | | |
| PUFA$$ (g) | 13.88 ± 3.76 | 0.2405 | 75.86 |
| *n*-3 Fatty acids (g) | 1.06 ± 1.06 | | |
| *n*-6 Fatty acids (g) | 10.80 ± 7.50 | | |
| Unhealthy fatty acids | | | |
| Trans fat (g) | 3.15 ± 3.75 | -0.0767 | 41.38 |
| Saturated fat (g) | 28.6 ± 8.0 | | |
| Cholesterol (mg) | 279.4 ± 51.2 | | |
| Dietary fiber (g) | 18.8 ± 4.9 | 0.4062 | 100.00 |
| Whole grains (g) | 62.74 ± 61.82 | 0.1007 | 58.62 |
| Refined grains (g) | 96.33 ± 82.90 | -0.1998 | 27.59 |
| Legumes (g) | 51.15 ± 53.8 | 0.1888 | 68.97 |
| Nuts and Seeds (g) | 1.57 ± 2.07 | 0.2652 | 89.66 |
| Oils (L) | 0.43 ± 0.23 | 0.0893 | 55.17 |
| Refined fats (g) | 4.57 ± 2.20 | -0.2734 | 17.24 |
| Soft drinks (L) | 0.37 ± 0.07 | -0.3693 | 3.45 |
| Salt (g) | 3.6 ± 1.2 | -0.2784 | 13.79 |
| Coffee/tea/waters (L) | 1.33 ± 0.52 | 0.1662 | 65.52 |
| Processed foods (g) | 186.67 ± 100 | -0.3083 | 6.9 |
| Sugars (g) | 97.85 ± 40 | -0.1782 | 31.03 |
| Fruits (g) | 183.4 ± 118.2 | 0.2491 | 82.76 |
| Vegetables (g) | 175.3 ± 86.1 | 0.2469 | 79.31 |
| Red meat (g) | 43.83 ± 23.29 | -0.2954 | 10.34 |
| Processed meat (g) | 17.00 ± 11.65 | -0.2448 | 20.69 |
| White meat (g) | 35 ± 25 | 0.1283 | 62.07 |
| Fish (g) | 92.43 ± 85.86 | 0.2497 | 86.21 |
| Dairy (g) | 113.5 ± 50.29 | 0.2081 | 72.41 |
| Diet drinks (mL) | 56.3 ± 46.2 | -0.1343 | 37.93 |
| Alcoholic drinks (L) | 0.38 ± 0.07 | -0.1618 | 34.48 |
| Vitamins and minerals | | | |
| Vitamin $B_{12}$ (μg) | 5.15 ± 2.70 | | |
| Vitamin $B_6$ (mg) | 1.47 ± 0.74 | | |
| Folic acid (μg) | 273.0 ± 70.7 | | |
| Thiamin (mg) | 1.70 ± 0.66 | | |
| Riboflavin (mg) | 1.70 ± 0.79 | | |
| Niacin (mg) | 25.90 ± 11.77 | | |
| Pantothenic acid (mg) | 5 ± 2 | | |
| Vitamin C (mg) | 118.2 ± 43.46 | | |
| Vitamin A (RE) | 983.9 ± 518.6 | 0.2776 | 93.10 |
| Vitamin D (μg) | 6.26 ± 2.21 | | |
| Vitamin E (mg) | 8.73 ± 1.49 | | |
| Vitamin K (μg) | 125 ± 50 | | |

| | | | |
|---|---|---|---|
| Calcium (mg) | 684 ± 211 | | |
| Se (µg) | 67.0 ± 25.1 | | |
| Zn (mg) | 9.84 ± 2.19 | | |
| Fe (mg) | 13.35 ± 3.71 | | |
| Mg (mg) | 310.1 ± 139.4 | | |
| Copper (mg) | 1.6 ± 0.4 | | |
| Manganese (mg) | 3.0 ± 1.0 | | |
| Secondary plant metabolites | | | |
| B-carotene (µg) | 3718 ± 1720 | | |
| A-carotene (µg) | 350 ± 200 | | |
| B-cryptoxanthin (µg) | 250 ± 150 | 0.34235 | 96.55 |
| Lycopene (µg) | 4433 ± 2267 | | |
| Lutein/zeaxanthin (µg) | 900 ± 296 | | |
| Caffeine (mg) | 805 ± 667 | | |

* From the world food consumption database, Whole Grain Consumption Worldwide | The Whole Grains Council, Whole grain intake across European countries | Knowledge for policy, Hydrogenated Fat Market Size, Share, Trends, Forecast To 2032, Soda Consumption by Country 2025, Salt Consumption by Country 2025, RF_SDMEST, Estimated intake of sodium in adults aged 25 and older (g/day), International Coffee Organization |, Statistics | FAO | Food and Agriculture Organization of the United Nations, Annual soft drink consumption volume EU 2013-2023 | Statista, Per capita alcohol consumption worldwide 2024, by country | Statista, EFSA: Food consumption data and Global Dietary Database (GDD) [6-21]. $Multi-unsaturated fatty acids, $$Poly-unsaturated fatty acids.

**Supplementary Table S3.** Association (ORs and 95%CIs) between different WDI estimation methods and various MetS definitions in crude logistic regression models.

| Calculation methods* | WHO | p-value | ATPIII | p-value | IDF | p-value |
|---|---|---|---|---|---|---|
| WDI-G | 0.234 (0.167, 0.330) | < 0.001 | 0.342 (0.267, 0.439) | < 0.001 | 0.323 (0.251, 0.415) | < 0.001 |
| WDI-GC | 0.484 (0.408, 0.574) | < 0.001 | 0.585 (0.517, 0.622) | < 0.001 | 0.568 (0.501, 0.644) | < 0.001 |
| WDI-GS | 0.747 (0.697, 0.800) | < 0.001 | 0.806 (0.767, 0.847) | < 0.001 | 0.797 (0.757, 0.838) | < 0.001 |
| WDI-P | 0.002 (0.000421, 0.007) | < 0.001 | 0.010 (0.004, 0.028) | < 0.001 | 0.010 (0.004, 0.029) | < 0.001 |
| WDI-PC | 0.041 (0.021, 0.082) | < 0.001 | 0.101 (0.061, 0.169) | < 0.001 | 0.102 (0.061, 0.171) | < 0.001 |
| WDI-PS | 0.733 (0.685, 0.784) | < 0.001 | 0.800 (0.761, 0.841) | < 0.001 | 0.801 (0.726, 0.742) | < 0.001 |
| WDI-I | 0.952 (0.774, 1.173) | 0.646 | 0.995 (0.861, 1.150) | 0.945 | 1.038 (0.896, 1.203) | 0.618 |
| WDI-FG | 0.266 (0.202, 0.350) | < 0.001 | 0.393 (0.322, 0.479) | < 0.001 | 0.388 (0.318, 0.474) | < 0.001 |

* For the detailed calculation methods, please see the methods and materials section (in all methods, higher scores indicate lower adherence to WDPs).

WDI: Westernized diet index, WDI-G: WDI global, WDI-GC: WDI global centralized, WDI-GS: WDI global standardized, WDI-P: WDI population, WDI-PC: WDI population centralized, WDI-PS: WDI population standardized, WDI-I: WDI individuald, WDI-FG: WDI food groups, OR: odds ratio, CI: confidence interval, MetS: metabolic syndrome, WHO: World Health Organization, ATPIII: Adult Treatment Panel III, IDF: international diabetes federation.

**Supplementary Table S4.** Association (ORs and 95%CIs) between different WDI estimation methods and various MetS definitions in logistic regression models adjusted for age and sex.

| Calculation methods* | WHO | p-value | ATPIII | p-value | IDF | p-value |
|---|---|---|---|---|---|---|
| WDI-G | 0.201 (0.140, 0.290) | < 0.001 | 0.304 (0.234, 0.395) | < 0.001 | 0.268 (0.204, 0.351) | < 0.001 |
| WDI-GC | 0.449 (0.374, 0.538) | < 0.001 | 0.551 (0.483, 0.628) | < 0.001 | 0.517 (0.452, 0.592) | < 0.001 |
| WDI-GS | 0.724 (0.673, 0.779) | < 0.001 | 0.787 (0.746, 0.829) | < 0.001 | 0.767 (0.726, 0.810) | < 0.001 |
| WDI-P | 0.0012 (0.0003, 0.0055) | < 0.001 | 0.008 (0.003, 0.025) | < 0.001 | 0.006 (0.002, 0.020) | < 0.001 |
| WDI-PC | 0.035 (0.017, 0.075) | < 0.001 | 0.092 (0.053, 0.159) | < 0.001 | 0.080 (0.045, 0.142) | < 0.001 |
| WDI-PS | 0.722 (0.672, 0.777) | < 0.001 | 0.793 (0.751, 0.836) | < 0.001 | 0.782 (0.740, 0.827) | < 0.001 |
| WDI-I | 0.634 (0.513, 0.783) | < 0.001 | 0.630 (0.541, 0.733) | < 0.001 | 0.579 (0.494, 0.678) | < 0.001 |
| WDI-FG | 0.276 (0.204, 0.371) | < 0.001 | 0.428 (0.345, 0.531) | < 0.001 | 0.412 (0.329, 0.516) | < 0.001 |

* For the detailed calculation methods, please see the methods and materials section (in all methods, higher scores indicate lower adherence to WDPs).

WDI: Westernized diet index, WDI-G: WDI global, WDI-GC: WDI global centralized, WDI-GS: WDI global standardized, WDI-P: WDI population, WDI-PC: WDI population centralized, WDI-PS: WDI population standardized, WDI-I: WDI individual, WDI-FG: WDI food groups, OR: odds ratio, CI: confidence interval, MetS: metabolic syndrome, WHO: World Health Organization, ATPIII: Adult Treatment Panel III, IDF: International Diabetes Federation.

**Supplementary Table S5**. Association (β and 95%CIs) between different WDI estimation methods* and various metabolic (bio)markers in crude linear regression models.

| Bio(marker) | WDI-G | p-value | WDI-GC | p-value | WDI-GS | p-value | WDI-P | p-value | WDI-PC | p-value | WDI-PS | p-value | WDI-I | p-value | WDI-FG | p-value |
|---|---|---|---|---|---|---|---|---|---|---|---|---|---|---|---|---|
| WC (cm) | -8.869 (-10.035, -7.702) | < 0.001 | -4.434 (-5.017, -3.851) | < 0.001 | -1.785 (-2.020, -1.550) | < 0.001 | -35.410 (-40.238, -30.582) | < 0.001 | -17.705 (-20.119, -15.291) | < 0.001 | -1.723 (-1.958, -14.88) | < 0.001 | -1.776 (-2.446, -1.106) | < 0.001 | -6.258 (-7.166, -5.350) | < 0.001 |
| HC (cm) | -6.282 (-7.162, -5.402) | < 0.001 | -3.141 (-3.581, -2.701) | < 0.001 | -1.264 (-1.441, -1.087) | < 0.001 | -23.317 (-26.965, -19.669) | < 0.001 | -11.658 (-13.482, -9.834) | < 0.001 | -1.135, -1.312, -0.957) | < 0.001 | -2.068 (-2.572, -1.564) | < 0.001 | -3.868 (-4.554, -3.182) | < 0.001 |
| WHR | -0.031 (-0.037, -0.025) | < 0.001 | -0.016 (-0.019, -0.012) | < 0.001 | -0.006 (-0.008, -0.005) | < 0.001 | -0.140 (-0.167, -0.114) | < 0.001 | -0.070 (-0.083, -0.057) | < 0.001 | -0.007 (-0.008, -0.006) | < 0.001 | 0.002 (-0.002, 0.005) | 0.808 | -0.027 (-0.032, -0.022) | < 0.001 |
| BMI (kg/m²) | -3.658 (-4.135, -3.181) | < 0.001 | -1.829 (-2.068, -1.590) | < 0.001 | -0.736 (-0.832, -0.640) | < 0.001 | -13.863 (-15.841, -11.885) | < 0.001 | -6.931 (-7.920, -5.942) | < 0.001 | -0.675 (-0.771, -0.578) | < 0.001 | -0.829 (-1.103, -0.555) | < 0.001 | -2.441 (-2.813, -2.069) | < 0.001 |
| DBP (mmHg) | -4.222 (-5.418, -3.026) | < 0.001 | -2.111 (-2.709, -1.513) | < 0.001 | -0.850 (-1.090, -0.609) | < 0.001 | -22.646 (-27.585, -17.706) | < 0.001 | -11.232 (-13.792, -8.853) | < 0.001 | -1.102 (-1.342, -0.862) | < 0.001 | -2.129 (-2.809, -1.448) | < 0.001 | -3.616 (-4.545, -2.687) | < 0.001 |
| SBP (mmHg) | -5.902 (-7.757, -4.047) | < 0.001 | -2.951 (-3.879, -2.024) | < 0.001 | -1.188 (-1.561, -0.814) | < 0.001 | -32.389 (-40.049, -24.729) | < 0.001 | -16.194 (-20.024, -12.364) | < 0.001 | -1.576 (-1.949, -1.203) | < 0.001 | -1.579 (-2.635, -0.523) | 0.003 | -5.416 (-6.855, -3.976) | < 0.001 |
| PR (bpm) | -2.221 (-3.295, -1.147) | < 0.001 | -1.111 (-1.648, -0.573) | < 0.001 | -0.447 (-0.663, -0.231) | < 0.001 | -7.298 (-11.743, -2.854) | < 0.001 | -3.649 (-5.871, -1.427) | < 0.001 | -0.355 (-0.571, -0.139) | < 0.001 | 0.527 (-0.084, 1.138) | 0.091 | -1.356 (-2.191, -0.521) | 0.001 |
| MAP (mmHg) | -4.782 (-6.130, -3.434) | < 0.001 | -2.391 (-3.065, -1.717) | < 0.001 | -0.962 (-1.234, -0.691) | < 0.001 | -25.893 (-31.457, -20.329) | < 0.001 | -12.947 (-15.729, -10.165) | < 0.001 | -1.260 (-1.531, -0.989) | < 0.001 | -1.946 (-2.713, -1.179) | < 0.001 | -4.216 (-5.262, -3.170) | < 0.001 |
| FBG (mg/dL) | -10.675 (-13.626, -7.723) | < 0.001 | -5.337 (-6.813, -3.862) | < 0.001 | -2.148 (-2.742, -1.554) | < 0.001 | -59.095 (-68.282, -43.907) | < 0.001 | -28.047 (-34.141, -21.954) | < 0.001 | -2.730 (-3.232, -2.137) | < 0.001 | -0.891 (-2.573, 0.791) | 0.299 | -10.521 (-12.810, -8.232) | < 0.001 |
| TG (mg/dL) | -39.385 (-47.601, -31.169) | < 0.001 | -19.693 (-23.801, -15.584) | < 0.001 | -7.926 (-9.580, -6.273) | < 0.001 | -125.403 (-159.447, -91.358) | < 0.001 | -62.701 (-79.724, -45.679) | < 0.001 | -6.102 (-7.759, -4.445) | < 0.001 | -15.764 (-20.445, -11.083) | < 0.001 | -17.738 (-24.139, -11.337) | < 0.001 |
| Cholesterol (mg/dL) | -5.951 (-9.854, -2.048) | < 0.001 | -2.976 (-4.927, -1.024) | < 0.001 | -1.198 (-1.983, -0.412) | < 0.001 | -20.220 (-36.365, -4.076) | < 0.001 | -10.110 (-18.182, -2.038) | < 0.001 | -0.984 (-1.770, -0.198) | < 0.001 | 0.380 (-1.839, 2.599) | 0.737 | -3.569 (-6.601, -0.0537) | 0.021 |
| LDL-c (mg/dL) | 1.316 (-0.89, 2.920) | 0.108 | 0.658 (-0.144, 1.460) | 0.108 | 0.265 (-0.058, 0.588) | 0.108 | -5.726 (-12.362, 0.910) | 0.091 | -2.863 (-6.181, 0.455) | 0.091 | -0.279 (-0.602, 0.044) | 0.091 | -0.843 (-1.755, 0.069) | 0.070 | -0.102 (-1.349, 1.144) | 0.872 |
| HDL-c (mg/dL) | 0.594 (-2.692, 3.880) | 0.723 | 0.297 (-1.346, 1.940) | 0.723 | 0.120 (-0.542, 0.781) | 0.723 | 10.501 (-3.088, 24.090) | 0.130 | 5.250 (-1.544, 12.045) | 0.130 | 0.511 (-0.150, 1.172) | 0.130 | 4.409 (2.544, 6.275) | < 0.001 | 0.055 (-2.498, 2.607) | 0.967 |

* For the detailed calculation methods, please see the methods and materials section (in all methods, higher scores indicate lower adherence to WDPs).

WDI: Westernized diet index, WDI-G: WDI global, WDI-GC: WDI global centralized, WDI-GS: WDI global standardized, WDI-P: WDI population, WDI-PC: WDI population centralized, WDI-PS: WDI population standardized, WDI-I: WDI individuald, WDI-FG: WDI food groups, β: beta, CI: confidence interval, MetS: metabolic syndrome, DBP: diastolic blood pressure, SBP: systolic blood pressure, PR: pulse rate, bpm: beats per minute, FBG: fasting blood glucose, TG: triglycerides, LDL-c: low-density lipoprotein cholesterol, HDL-c: high-density lipoprotein cholesterol.

**Supplementary Table S6.** Association (β and 95%CIs) between different WDI estimation methods* and various metabolic (bio)markers in linear regression models adjusted for age and sex.

| Bio(marker) | WDI-G | R/ARS | p-value | WDI-GC | R/ARS | p-value | WDI-GS | R/ARS | p-value | WDI-P | R/ARS | p-value | WDI-PS | R/ARS | p-value | WDI-PS | R/ARS | p-value | WDI-I | R/ARS | p-value | WDI-FG | R/ARS | p-value |
|---|---|---|---|---|---|---|---|---|---|---|---|---|---|---|---|---|---|---|---|---|---|---|---|---|
| WC (cm) | -8.973 (-10.088, -7.857) | 0.327/0.106 | < 0.001 | -4.486 (-5.044, -3.928) | 0.327/0.106 | < 0.001 | -1.806 (-2.030, -1.581) | 0.327/0.106 | < 0.001 | -33.748 (-38.390, -29.106) | 0.320/0.102 | < 0.001 | -16.874 (-19.195, -14.553) | 0.320/0.102 | < 0.001 | -1.642 (-1.868, -1.416) | 0.320/0.102 | < 0.001 | -4.312 (-4.967, -3.656) | 0.315/0.099 | < 0.001 | -5.237 (-6.115, -4.359) | 0.310/0.096 | < 0.001 |
| HC (cm) | -6.507 (-7.361, -5.653) | 0.280/0.078 | < 0.001 | -3.253 (-3.680, -2.826) | 0.280/0.078 | < 0.001 | -1.309 (-1.481, -1.138) | 0.280/0.078 | < 0.001 | -25.185 (-28.735, -21.635) | 0.275/0.075 | < 0.001 | -12.593 (-14.368, -10.818) | 0.275/0.075 | < 0.001 | -1.226 (-1.398, -1.053) | 0.275/0.075 | < 0.001 | -3.333 (-3.834, -2.832) | 0.271/0.073 | < 0.001 | -3.952 (-4.623, -3.280) | 0.264/0.070 | < 0.001 |
| WHR | -0.030 (-0.036, -0.024) | 0.401/0.160 | < 0.001 | -0.015 (-0.018, -0.012) | 0.401/0.160 | < 0.001 | -0.006 (-0.007, -0.005) | 0.401/0.160 | < 0.001 | -0.106 (-0.130, -0.081) | 0.398/0.158 | < 0.001 | -0.053 (-0.065, -0.041) | 0.398/0.158 | < 0.001 | -0.005 (-0.006, -0.004) | 0.398/0.158 | < 0.001 | -0.013 (-0.016, -0.009) | 0.395/0.156 | < 0.001 | -0.016 (-0.020, -0.011) | 0.395/0.156 | < 0.001 |
| BMI (kg/m²) | -3.757 (-4.216, -3.299) | 0.316/0.099 | < 0.001 | -1.879 (-2.108, -1.650) | 0.316/0.099 | < 0.001 | -0.756 (-0.848, -0.664) | 0.316/0.099 | < 0.001 | -14.170 (-16.078, -12.263) | 0.309/0.095 | < 0.001 | -7.085 (-8.039, -6.131) | 0.309/0.095 | < 0.001 | -0.690 (-0.782, -0.597) | 0.309/0.095 | < 0.001 | -1.769 (-2.038, -1.499) | 0.0302/0.091 | < 0.001 | -2.261 (-2.622, -1.900) | 0.300/0.089 | < 0.001 |
| DBP (mmHg) | -3.990 (-5.162, -2.817) | 0.211/0.044 | < 0.001 | -1.995 (-2.281, -1.408) | 0.211/0.044 | < 0.001 | -0.803 (-1.039, -0.567) | 0.211/0.044 | < 0.001 | -18.491 (-23.355, -13.626) | 0.214/0.045 | < 0.001 | -9.245 (-11.678, -6.813) | 0.214/0.045 | < 0.001 | -0.900 (-1.136, -0.663) | 0.214/0.045 | < 0.001 | -2.852 (-3.538, -2.167) | 0.216/0.046 | < 0.001 | -2.584 (-3.502, -1.666) | 0.208/0.043 | < 0.001 |
| SBP (mmHg) | -5.239 (-6.970, -3.508) | 0.365/0.133 | < 0.001 | -2.620 (-3.485, -1.754) | 0.365/0.133 | < 0.001 | -1.054 (-1.403, -0.706) | 0.365/0.133 | < 0.001 | -20.529 (-27.716, -13.343) | 0.365/0.133 | < 0.001 | -10.265 (-13.858, -6.971) | 0.365/0.133 | < 0.001 | -0.999 (-1.349, -0.649) | 0.365/0.133 | < 0.001 | -3.242 (-4.255, -2.229) | 0.366/0.133 | < 0.001 | -2.485 (-3.841, -1.129) | 0.362/0.131 | < 0.001 |
| PR (bpm) | -2.363 (-3.407, -1.320) | 0.244/0.059 | < 0.001 | -1.182 (-1.703, -0.663) | 0.244/0.059 | < 0.001 | -0.476 (-0.686, -0.266) | 0.244/0.059 | < 0.001 | -6.905 (-11.273, -2.573) | 0.242/0.058 | < 0.001 | -3.453 (-5.619, -1.287) | 0.242/0.058 | < 0.001 | -0.336 (-0.547, -0.125) | 0.242/0.058 | < 0.001 | -1.258 (-1.868, -0.647) | 0.243/0.059 | < 0.001 | -0.752 (-1.569, 0.065) | 0.241/0.058 | 0.071 |
| MAP (mmHg) | -4.406 (-5.699, -3.113) | 0.291/0.085 | < 0.001 | -2.203 (-2.850, -1.557) | 0.291/0.085 | < 0.001 | -0.887 (-1.147, -0.627) | 0.291/0.085 | < 0.001 | -19.170 (-24.536, -13.804) | 0.292/0.085 | < 0.001 | -9.585 (-12.268, -6.902) | 0.292/0.085 | < 0.001 | -0.933 (-1.194, -0.672) | 0.292/0.085 | < 0.001 | -2.982 (-3.738, -2.226) | 0.294/0.086 | < 0.001 | -2.551 (-3.564, -1.538) | 0.288/0.083 | < 0.001 |
| FBG (mg/dL) | -10.192 (-13.080, -7.303) | 0.219/0.048 | < 0.001 | -5.096 (-6.540, -3.651) | 0.219/0.048 | < 0.001 | -2.051 (-2.632, -1.470) | 0.219/0.048 | < 0.001 | -45.919 (-57.904, -33.935) | 0.221/0.049 | < 0.001 | -22.960 (-28.952, -16.967) | 0.221/0.049 | < 0.001 | -2.234 (-2.818, -1.651) | 0.221/0.049 | < 0.001 | -3.317 (-5.011, -1.624) | 0.211/0.044 | < 0.001 | -7.750 (-10.011, -5.490) | 0.218/0.047 | < 0.001 |
| TG (mg/dL) | -39.055 (-47.265, -30.844) | 0.107/0.011 | < 0.001 | -19.527 (-23.633, -15.422) | 0.107/0.011 | < 0.001 | -7.860 (-9.512, -6.207) | 0.107/0.011 | < 0.001 | -124.614 (-158.755, -90.473) | 0.088/0.007 | < 0.001 | -62.307 (-79.377, -45.237) | 0.088/0.007 | < 0.001 | -6.064 (-7.725, -4.402) | 0.088/0.007 | < 0.001 | -14.207 (-19.025, -9.390) | 0.077/0.006 | < 0.001 | -18.369 (-24.811, -11.927) | 0.075/0.005 | < 0.001 |
| Cholesterol (mg/dL) | -5.889 (-9.734, -2.045) | 0.177/0.031 | 0.003 | -2.945 (-4.867, -1.022) | 0.177/0.031 | 0.003 | -1.185 (-1.959, -0.411) | 0.177/0.031 | 0.003 | -13.055 (-29.017, 2.908) | 0.175/0.030 | 0.109 | -6.527 (-14.509, 1.454) | 0.175/0.030 | 0.109 | -0.635 (-1.412, 0.141) | 0.175/0.030 | 0.109 | -4.127 (-6.376, -1.878) | 0.178/0.031 | < 0.001 | -0.758 (-3.767, 2.251) | 0.174/0.030 | 0.622 |
| LDL-c (mg/dL) | 1.216 (-0.351, 2.783) | 0.217/0.047 | 0.128 | 0.608 (-0.176, 1.391) | 0.217/0.047 | 0.128 | 0.245 (-0.071, 0.560) | 0.217/0.047 | 0.128 | -3.796 (-10.301, 2.709) | 0.217/0.047 | 0.253 | -1.898 (-5.150, 1.355) | 0.217/0.047 | 0.253 | -0.185 (-0.501, 0.132) | 0.217/0.047 | 0.253 | -3.371 (-4.286, -2.456) | 0.228/0.052 | < 0.001 | 1.850 )-0.702, 4.402) | 0.124/0.015 | 0.155 |
| HDL-c (mg/dL) | 0.689 (-2.573, 3.951) | 0.123/0.015 | 0.679 | 0.345 (-1.278, 1.976) | 0.123/0.015 | 0.679 | 0.139 (-0.581, 0.795) | 0.123/0.015 | 0.679 | 15.561 (2.024, 29.098) | 0.125/0.015 | 0.024 | 7.780 (1.012, 14.549) | 0.125/0.015 | 0.024 | 0.757 (0.098, 1.416) | 0.125/0.015 | 0.024 | 2.126 (0.217, 4.034) | 0.125/0.015 | 0.029 | 1.035 (-0.191, 2.261) | 0.217/0.047 | 0.098 |

* For the detailed calculation methods, please see the methods and materials section (in all methods, higher scores indicate lower adherence to WDPs).

WDI: Westernized diet index, WDI-G: WDI global, WDI-GC: WDI global centralized, WDI-GS: WDI global standardized, WDI-P: WDI population, WDI-PC: WDI population centralized, WDI-PS: WDI population standardized, WDI-I: WDI individuald, WDI-FG: WDI food groups, β: beta, CI: confidence interval, MetS: metabolic syndrome, DBP: diastolic blood pressure, SBP: systolic blood pressure, PR: pulse rate, bpm: beats per minute, FBG: fasting blood glucose, TG: triglycerides, LDL-c: low-density lipoprotein cholesterol, HDL-c: high-density lipoprotein cholesterol, R: R-squared, ARS: adjusted R-squared.

**Supplementary Table S7**. Final cluster centers for health indicators and WDI, showing distinct patterns in healthy and unhealthy clusters.

| Health indicators (biomarkers) | Cluster 1 (Healthy) | Cluster 2 (Unhealthy) |
|---|---|---|
| WC (cm) | 92.53 | 98.14 |
| HC (cm) | 99.24 | 101.89 |
| WHR | 0.93 | 0.96 |
| BMI (kg/m$^2$) | 25.42 | 27.56 |
| DPB (mmHg) | 74 | 78 |
| SPB (mmHg) | 111 | 116 |
| PR (bpm) | 74 | 76 |
| MAP (mmHg) | 86.28 | 90.36 |
| FBG (mg/dL) | 91.71 | 101.51 |
| TG (mg/dL) | 109.21 | 290.84 |
| Cholesterol (mg/dL) | 181.24 | 217.32 |
| LDL-c (mg/dL) | 52.31 | 45.51 |
| HDL-c (mg/dL) | 107.06 | 113.63 |
| WDI-G* | -0.13 | -0.18 |
| WDI-P* | -0.01 | -0.02 |
| WDI-FG* | 0.00 | -0.03 |

* were entered separately in the K-means analysis.

DBP: diastolic blood pressure, SBP: systolic blood pressure, PR: pulse rate, bpm: beats per minute, FBG: fasting blood glucose, TG: triglycerides, LDL-c: low-density lipoprotein cholesterol, HDL-c: high-density lipoprotein cholesterol, WDI: Westernized diet index, WDI-G: WDI global, WDI-P: WDI population, WDI-FG: WDI food groups.

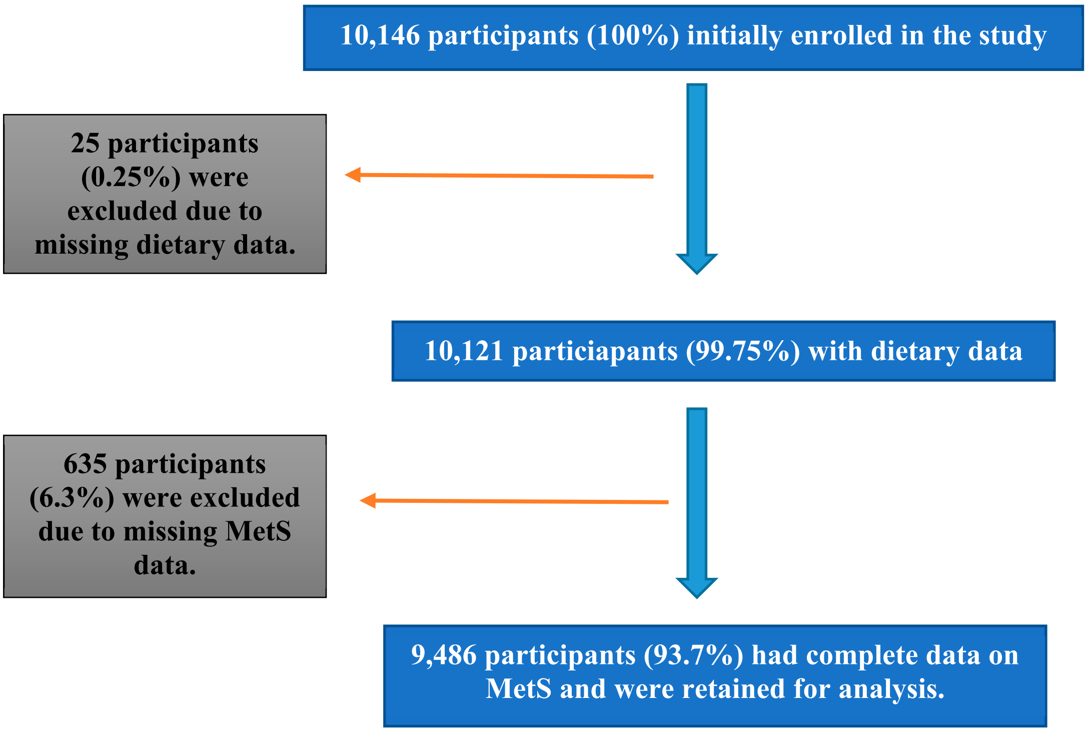


**Supplementary Figure S1**. Flow diagram of participants through the study.

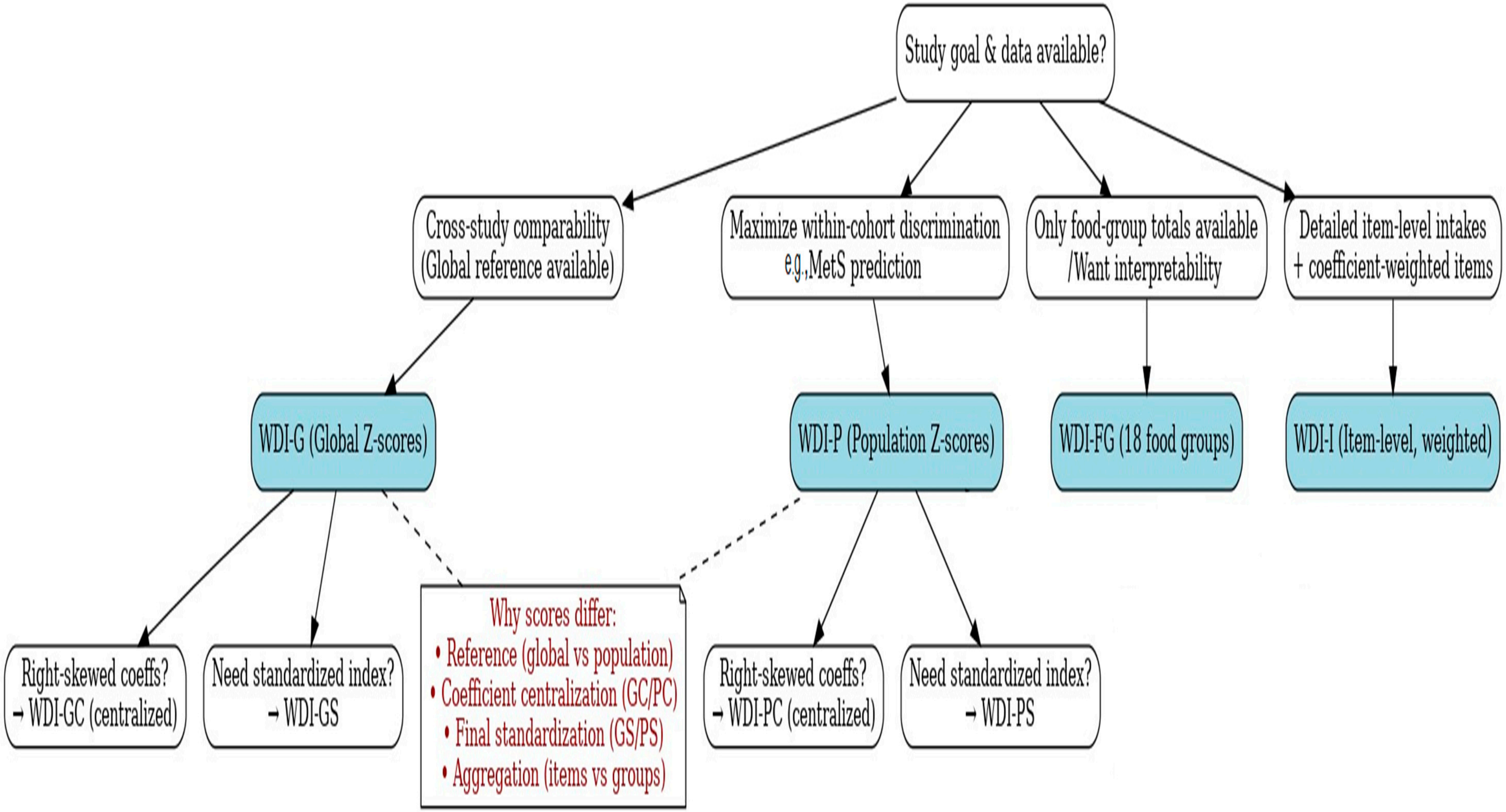


**Supplementary Figure S2.** Decision Guide for WDI: Illustrating When and Why to Select Different WDI Calculation Methods.

WDI: Westernized diet index, WDI-G: WDI global, WDI-GC: WDI global centralized, WDI-GS: WDI global standardized, WDI-P: WDI population, WDI-PC: WDI population centralized, WDI-PS: WDI population standardized, WDI-I: WDI individuald, WDI-FG: WDI food groups.

## References


1. Alberti, K.G.M.M.; Zimmet, P.Z. Definition, diagnosis and classification of diabetes mellitus and its complications. Part 1: diagnosis and classification of diabetes mellitus. Provisional report of a WHO consultation. *Diabetic medicine* **1998**, *15*, 539-553.
2. Grundy, S.M.; Cleeman, J.I.; Merz, C.N.B.; Brewer Jr, H.B.; Clark, L.T.; Hunninghake, D.B.; Pasternak, R.C.; Smith Jr, S.C.; Stone, N.J. Implications of recent clinical trials for the national cholesterol education program adult treatment panel III guidelines. *Circulation* **2004**, *110*, 227-239.
3. Detection, N.C.E.P.E.P.o.; Adults, T.o.H.B.C.i. *Third report of the National Cholesterol Education Program (NCEP) Expert Panel on detection, evaluation, and treatment of high blood cholesterol in adults (Adult Treatment Panel III)*; The Program: 2002.
4. Alberti, K.G.; Zimmet, P.; Shaw, J. Metabolic syndrome--a new world-wide definition. A Consensus Statement from the International Diabetes Federation. *Diabet Med* **2006**, *23*, 469-480, doi:10.1111/j.1464-5491.2006.01858.x.
5. Cifuentes, M.; Hejazi, Z.; Vahid, F.; Bohn, T. Designing and Developing a Population/Literature-Based Westernized Diet Index (WDI) and Its Relevance for Cardiometabolic Health. In *Nutrients*, 2025; Vol. 17.
6. Micha, R.; Khatibzadeh, S.; Shi, P.; Andrews, K.G.; Engell, R.E.; Mozaffarian, D. Global, regional and national consumption of major food groups in 1990 and 2010: a systematic analysis including 266 country-specific nutrition surveys worldwide. *BMJ Open* **2015**, *5*, e008705, doi:10.1136/bmjopen-2015-008705.
7. Skórska, K.B.; Grajeta, H.; Zabłocka-Słowińska, K.A. Frequency of legume consumption related to sociodemographic factors, health status and health-related variables among surveyed adults from Poland. *Public Health Nutr* **2021**, *24*, 1895-1905, doi:10.1017/s1368980020002116.
8. Hughes, J.; Pearson, E.; Grafenauer, S. Legumes-A Comprehensive Exploration of Global Food-Based Dietary Guidelines and Consumption. *Nutrients* **2022**, *14*, doi:10.3390/nu14153080.
9. Jenab, M.; Sabaté, J.; Slimani, N.; Ferrari, P.; Mazuir, M.; Casagrande, C.; Deharveng, G.; Tjønneland, A.; Olsen, A.; Overvad, K. Consumption and portion sizes of tree nuts, peanuts and seeds in the European Prospective Investigation into Cancer and Nutrition (EPIC) cohorts from 10 European countries. *British Journal of Nutrition* **2006**, *96*, S12-S23.
10. Canton, H. Food and agriculture organization of the United Nations—FAO. In *The Europa directory of international organizations 2021*, Routledge: 2021; pp. 297-305.
11. Silva, T.J.; Barrera-Arellano, D.; Ribeiro, A.P.B. Margarines: Historical approach, technological aspects, nutritional profile, and global trends. *Food Res Int* **2021**, *147*, 110486, doi:10.1016/j.foodres.2021.110486.
12. Górska-Warsewicz, H.; Rejman, K.; Laskowski, W.; Czeczotko, M. Butter, Margarine, Vegetable Oils, and Olive Oil in the Average Polish Diet. In *Nutrients*, 2019; Vol. 11.
13. Cormick, G.; Belizán, J.M. Calcium Intake and Health. In *Nutrients*, 2019; Vol. 11.
14. EFSA Panel on Dietetic Products, N.; Allergies. Scientific opinion on dietary reference values for copper. *EFSA Journal* **2015**, *13*, 4253.
15. Sadhra, S.S.; Wheatley, A.D.; Cross, H.J. Dietary exposure to copper in the European Union and its assessment for EU regulatory risk assessment. *Sci Total Environ* **2007**,

*374*, 223-234, doi:10.1016/j.scitotenv.2006.12.041.
16. EFSA Panel on Dietetic Products, N.; Allergies; Turck, D.; Bresson, J.L.; Burlingame, B.; Dean, T.; Fairweather-Tait, S.; Heinonen, M.; Hirsch-Ernst, K.I.; Mangelsdorf, I., et al. Dietary reference values for vitamin K. *EFSA Journal* **2017**, *15*, e04780.
17. Booth, S.L. Vitamin K: food composition and dietary intakes. *Food Nutr Res* **2012**, *56*, doi:10.3402/fnr.v56i0.5505.
18. Huang, Y.; Chen, Z.; Chen, B.; Li, J.; Yuan, X.; Li, J.; Wang, W.; Dai, T.; Chen, H.; Wang, Y., et al. Dietary sugar consumption and health: umbrella review. *Bmj* **2023**, *381*, e071609, doi:10.1136/bmj-2022-071609.
19. Kalmpourtzidou, A.; Eilander, A.; Talsma, E.F. Global Vegetable Intake and Supply Compared to Recommendations: A Systematic Review. *Nutrients* **2020**, *12*, doi:10.3390/nu12061558.
20. Cancer, I.A.f.R.o. Working Group on the Evaluation of Carcinogenic Risks to Humans Red Meat and Processed Meat. *IARC Monographs on the Evaluation of Carcinogenic Risks to Humans* **2018**, *114*.
21. Shivappa, N.; Steck, S.E.; Hurley, T.G.; Hussey, J.R.; Hébert, J.R. Designing and developing a literature-derived, population-based dietary inflammatory index. *Public Health Nutr* **2014**, *17*, 1689-1696, doi:10.1017/s1368980013002115.
